# Testcross performance and combining ability of early-medium maturing quality protein maize inbred lines in Eastern and Southern Africa

**DOI:** 10.1038/s41598-024-58816-y

**Published:** 2024-04-21

**Authors:** Addisalem Mebratu, Dagne Wegary, Adefris Teklewold, Amsal Tarekegne

**Affiliations:** 1 College of Agriculture and Natural Resource, Wolkite University, P.O. Box 07, Wolkite, Ethiopia; 2 International Maize and Wheat Improvement Center, P.O. Box MP163, Harare, Zimbabwe; 3 International Maize and Wheat Improvement Center, ILRI Campus, P.O. Box 5689, Addis Ababa, Ethiopia; 4 Zambia Seed Company Limited, P.O. Box 35441, Lusaka, Zambia

**Keywords:** Maize, Gene action, GGE biplot, Protein quality, QPM, Plant sciences, Plant breeding

## Abstract

Limited commercial quality protein maize (QPM) varieties with low grain yield potential are currently grown in Eastern and Southern Africa (ESA). This study was conducted to (i) assess the performance of single-cross QPM hybrids that were developed from elite inbred lines using line-by-tester mating design and (ii) estimate the general (GCA) and specific (SCA) combining ability of the QPM inbred lines for grain yield, agronomic and protein quality traits. One hundred and six testcrosses and four checks were evaluated across six environments in ESA during 2015 and 2016. Significant variations (*P* ≤ 0.01) were observed among environments, genotypes and genotype by environment interaction (GEI) for most traits evaluated. Hybrids H80 and H104 were the highest-yielding, most desirable, and stable QPM hybrids. Combining ability analysis showed both additive and non-additive gene effects to be important in the inheritance of grain yield. Additive effects were more important for agronomic and protein quality traits. Inbred lines L19 and L20 depicted desirable GCA effects for grain yield. Various other inbred lines with favorable GCA effects for agronomic traits, endosperm modification, and protein quality traits were identified. These inbred lines could be utilized for breeding desirable QPM cultivars. The QPM hybrids identified in this study could be commercialized after on-farm verification to replace the low-yielding QPM hybrids grown in ESA.

## Introduction

In sub-Saharan Africa (SSA), maize (*Zea mays* L.) is an important multipurpose crop with a significant contribution to food security, economic wellbeing and volume of grain production, covering more than half of the land allocated to cereals^1^. Millions of resource-poor smallholder farmers in several SSA countries rely on maize as their staple^2,3^. Besides, maize is a predominant source of calories (> 450 kcal capita^−1^ day^−1^) and protein (12 g capita^−1^ day^−1^) for resource-poor farmers in SSA^4^ with average per capita consumption of 50 kg year^−1^ in ESA. Maize consumption in Southern Africa is estimated to be more than 100 kg year^−1^ ^5^. Despite its major role as a staple in the SSA, normal endosperm maize is inherently deficient in two basic amino acids, namely, lysine and tryptophan^6–8^ resulting in malnutrition and nutrition insecurity for communities dependent on maize as their staple food^9^.

Reversing the share of zein to non-zein protein fraction has been the major focus of scientists to improve the nutritional quality of maize proteins^10^. This has been possible by identifying naturally existing *opaque-2* mutant alleles^11^ that alter the amino acid profile of maize endosperm protein and increase lysine and tryptophan levels as compared to the normal maize genotypes. The International Maize and Wheat Improvement Center (CIMMYT) bred new agronomically acceptable and nutritionally improved *opaque-2*- based maize germplasm which was later named as Quality Protein Maize (QPM)^12^. Due to its higher level of tryptophan and lysine content, QPM protein has a higher biological value (absorption and utilization of protein by the body) than the non-QPM maize genotypes^13^. Availability and utilization of amino acids from QPM proteins is 90% of amino acid composition of milk^14,15^. Feeding trials demonstrated a positive impact of  QPM on weight gain in poultry and pigs^16,17^.

QPM can significantly improve nutritional status in communities where maize is the primary protein source and alternative protein sources are scarce and unaffordable^6,10,18,19^. QPM breeding is, therefore, an affordable and sustainable approach to alleviate protein deficiency in areas where maize is the staple crop, as is the case in most ESA countries. For this purpose, CIMMYT’s maize breeding program in collaboration with national agricultural research systems (NARS) has been developing and deploying high-yielding QPM varieties to farmers^20–23^ in SSA. Positive impacts of QPM on nutritional status in children have also been documented^24^.

Despite the proven nutritional advantages of QPM cultivars, their uptake by farmers and seed companies has been lower than anticipated due to several factors^6,25–27^. Farmers frequently prioritize crop yield and productivity, potentially leading to reluctance to adopt QPM hybrids. Seed companies anticipate rapid adoption of the new generation QPM cultivars by farmers; however, uptake has been sluggish due to the invisible nature of the QPM trait, reluctance of grain traders to pay a premium price for QPM grain, lack of interest from maize food processors in marketing QPM as a premium product, lack of awareness about health advantages of QPM varieties and absence of government incentives to promote adoption through seed price subsidies^6,27^. In addition, the QPM trait governed by a recessive *opaque-2* gene and modifiers faces significant challenges due to contamination during seed and grain production, resulting in decreased levels of essential amino acids^26,27^. This poses a considerable hurdle for both seed companies and farmers, ultimately contributing to the slow uptake of QPM hybrids^25,27^.

The speed of genetic improvement in breeding crops depends on the relative importance of various gene effects. Various authors published results on the nature of gene actions governing grain yield, protein quality traits and endosperm modification traits in QPM germplasm; Bhatnagar et al*.*^28^, Machida et al.^29^ and Wegary et al.^30^ reported the preponderance of non-additive gene effects, whereas Musila et al.^21^, Nepir et al.^31^ and Abakemal et al.^32^ showed the importance of additive gene effects for grain yield. On the other hand, Jompuk et al.^33^, Wegary et al.^34^ and Njeri et al.^35^ underlined the importance of both additive and non-additive effects in inheriting these traits. The preponderances of additive gene effects for tryptophan content were reported by several investigators^29,31,34^. Wegary et al.^34^ reported the importance of both additive and non-additive gene effects in conferring the expression of protein content and kernel endosperm modification. Machida et al. ^29^, Nepir et al.^31^ and Abakemal et al.^32^ observed additive gene effects in controlling protein content and kernel endosperm modification more than the non-additive effects. Additive gene effects were shown to be more important than the non-additive effects in controlling protein quality index in QPM germplasm^29,31,34^.

Recently, CIMMYT has developed stress-resilient QPM inbred lines to enhance the nutritional impact of maize germplasm and increase the genetic gain of QPM under diverse smallholder farming conditions in ESA. Understanding the genetic potentials of the inbred lines in hybrid combinations would be of utmost importannce for their effective utilization. Therefore, combining ability analysis of the newly developed elite QPM inbred lines is a vital tool to identify and select the most desirable inbred lines for the development of high-yielding and nutritionally superior QPM cultivars adapted to the target environments in the region. The objectives of this study were to (i) assess the performance of single-cross QPM hybrids that were developed from elite inbred lines using line- by- tester mating design, and (ii) estimate GCA and SCA effects of the QPM inbred lines for grain yield and agronomic and protein quality traits.

## Materials and methods

### Experimental materials

One hundred and eight QPM testcrosses were obtained by crossing 27 early to medium maturing stress tolerant QPM inbred lines with four testers (Table 1) in a line-by-tester mating design^36^. The testcrosses were formed during the summer cropping season (November 2014–April 2015) in Harare, Zimbabwe. The inbred lines were selected through rigorous phenotypic evaluations in the breeding nurseries and screened for endosperm modification using a light table followed by biochemical analysis to determine tryptophan and protein content (Table 1). Most of the inbred lines were developed by converting popular normal inbred lines through backcross breeding^37^ and recycling elite QPM inbred lines. The testers included in this study are well-adapted and known to be useful in hybrid formation for tropical and sub-tropical mid-altitude environments.

**Table 1:** Grain yield and protein quality profiles of 27 early-medium maturing QPM inbred lines and four testers used in the study.

Parents	Name	Grain yield	Anthesis date	Protein content	Tryptophan concentration	Protein quality index
		t ha^−1^	days	–––––––––––– % –––––––––––-		
Line						
L1	TL156581	1.42	62	10.64	0.07	0.66
L2	TL156582	1.05	55	9.21	0.07	0.76
L3	TL148288	1.15	63	10.63	0.09	0.85
L4	TL156585	1.81	63	11.56	0.06	0.52
L5	TL156586	2.98	65	9.14	0.08	0.88
L6	TL156589	1.70	65	11.77	0.1	0.85
L7	TL156590	2.42	63	9.62	0.08	0.83
L8	TL156591	1.56	68	11.67	0.12	1.03
L9	TL148287	2.07	59	11.16	0.09	0.81
L10	TL116960	2.10	65	12.57	0.1	0.8
L11	TL116955	1.19	63	10.77	0.09	0.84
L12	TL13609	2.22	63	11.5	0.08	0.7
L13	TL156597	1.98	59	10.36	0.06	0.58
L14	TL156599	1.97	60	10.36	0.06	0.58
L15	TL156600	2.43	61	10.12	0.07	0.69
L16	TL156604	2.11	66	8.66	0.08	0.92
L17	TL155932	1.73	62	11.01	0.08	0.73
L18	TL155814	1.01	62	10.72	0.08	0.75
L19	TL156608	2.89	62	10.16	0.08	0.79
L20	TL156609	1.67	64	11.5	0.08	0.7
L21	VL05127	1.98	61	11.76	0.1	0.85
L22	TL135414	1.94	67	11.01	0.08	0.73
L23	TL155933	1.39	64	11.25	0.08	0.71
L24	TL156601	1.73	61	11.85	0.1	0.84
L25	TL156605	2.10	69	10.1	0.09	0.89
L26	VL06375	1.63	62	10.52	0.08	0.76
L27	TL156611	1.80	69	11.85	0.1	0.84
Inbred tester						
T1	TL156587	2.08	63	10.34	0.08	0.77
T2	VL05552	2.16	67	10.53	0.08	0.76
T3	TL148289	1.55	67	10.41	0.09	0.86
T4	TL149662	1.20	69	10.92	0.1	0.92
Mean		1.84	64	10.76	0.08	0.78

One hundred and six QPM testcross hybrids that had sufficient seed quantities were evaluated along with two commercial check hybrids (ZS261 and SC627). Two testcrosses (L18 × T1 and L18 × T2) could not be evaluated due to insufficient seeds. In addition, two location-specific popular standard checks were included for comparison. The commercial checks represent intermediate maturing QPM (ZS261) and non-QPM (SC627) hybrids widely grown in ESA. The standard checks used were AMH760Q (QPM) and AMH851 (non-QPM) at Ambo; BHQPY545 and BH546 at Bako; and SC403 and SC513 in the other environments. AMH760Q and AMH851 are three-way hybrids released for highland agro-ecologies of Ethiopia and widely adopted by farmers in country’s highland and transitional highland areas. BHQPY545 is a single cross yellow-grain QPM hybrid released for mid-altitude sub-humid regions of Ethiopia. BH546 is a high yielding and intermediate maturing non-QPM three-way hybrid released for mid-altitude sub-humid areas of the country. SC403 and SC513 are early and intermediate maturing, respectively, non-QPM hybrids released and marketed by Seed Co Ltd (Seed Co) in many SSA countries.

### Trial management and data collection

Field evaluations of 110 hybrids, involving 106 QPM experimental hybrids, two commercial and two standard check hybrids were carried out in 2015 and 2016 across six environments in ESA countries (Table 2). The test environments represent major maize-growing mega-environments of SSA^38^. Trials were conducted during main-cropping season May–November 2015 in Ethiopia and December 2015–May 2016 in Zimbabwe and Zambia.

**Table 2:** Description for the test locations used to evaluate the quality protein maize testcross hybrids.

Location code	Location	Country	Year	Co-ordinates	Rainfall (mm)	Temperature (°C)		Altitude	Fertilization			Grain yield (t ha^-1^)	
								(*m.a.s.l.*)	(kg ha^-1^)				
						Min	Max		N	P_2_O_5_	K	Mean ± SE	Range
BK	Bako	Ethiopia	2015	9^o^06'N and 37^o^09'E	944.4	12.3	29.8	1650	92	69	0	6.85 ± 0.91	3.23–10.18
AM	Ambo	Ethiopia	2015	8^o^57'N and 38^o^07'E	1050	10.4	26.3	2225	166	69	0	6.19 ± 0.86	2.57–9.23
GW	Gwebi	Zimbabwe	2016	17°13'S and 31°E	637	4.9	26.2	1406	166	56	24	6.53 ± 0.98	1.92–8.97
GL	Glendale	Zimbabwe	2016	17°31'S and 31°3'E	669	7.4	28.1	1250	166	56	24	5.88 ± 1.79	1.95–10.84
MP	Mpongwe	Zambia	2016	13°32’S and 28°03’E	1500	NA	NA	1300	208	35	21	6.46 ± 0.87	2.22–8.34
CH	Chisumbanje	Zimbabwe	2016	20°48’S and 32°14’E	455	NA	NA	415	166	56	24	5.25 ± 0.98	1.60–7.48

*m.a.s.l.*, Meters above sea level; NA, Not available.

The trials were conducted in each location using a 5 × 22 alpha lattice (0, 1) design^39^ with two replicates. The entries were hand-planted in single-row plots of 4.8 m length at Bako, 4.25 m at Ambo and 4 m in the other environments. The spacing used was 0.75 m between rows, and 0.25 m between hills in all environments except at Bako, where 0.3 m spacing between hills was used. Initially, two seeds were planted per hill and later thinned to one plant to achieve the desired plant densities of 44,444 plants ha^−1^ at Bako, and 53,333 plants ha^−1^ in all the other environments. Different fertilizer rates were applied based on site-specific recommendations (Table 2). Standard cultural practices recommended for growing maize were followed in all environments. Weeds were controlled using herbicides and hand weeding following the standard procedure of respective environments.

Data were recorded for grain yield (GY), number of days to anthesis (DA) and silking (DS), and number of ears per plant (EPP) following standard procedure used in maize characterization^40,41^. Plant height (PH) and ear height (EH) were measured as the average of 10 randomly sampled plants following the procedure. Endosperm modification (MOD) was scored on a light-table (1–5 scale) at Ambo, Ethiopia and Gwebi, Zimbabwe as described by Vivek et al.^37^ from 100 kernels randomly sampled from bulk grains from each plot.

### Laboratory analysis

Analysis of protein and amino acid content was conducted at CIMMYT’s Cereal Quality Laboratory in Mexico from 25 homogenized uniform-sized grain samples shelled and bulked from the middle part of 2–3 sib-mated (full-sib) ears as described hereafter. Tryptophan concentration (%) in whole-grain maize flour was determined by the colorimetric method based on glyoxylic acid^42^. The protein content was determined from the percent of nitrogen analyzed following the Micro Kjeldahl method and optimized by Villegas et al.^43^. Protein index (%) was calculated as described by Vivek et al.^37^ to compare the relative nutritional values of the hybrids.

### Statistical analysis

Analysis of variance for individual environments was conducted with the PROC MIXED procedure in SAS version 9.3 ^44^, considering genotypes as fixed effects and replications and blocks within replications as random effects. A combined analysis of variance was conducted for traits that showed significant differences among genotypes at individual environments with PROC GLM in the SAS computer package, using a RANDOM statement with the TEST option. Standard check hybrids were excluded from combined analysis since variable standard checks were used in different environments. In the combined analysis, the significance of genotype and environment mean squares were tested using the GEI mean square, whereas the GEI was tested against the pooled error.

The variation due to genotypes and GEI for GY was examined using a GGE biplot based on singular value decomposition (SVD) of the first two principal components^45^. The GGE biplot analysis with GenStat^®^ Release version 17.1 statistical software^46^ generated the ‘which-won-where’ and ‘mean versus stability’ biplot graphs. Mean values of the top-yielding 25 hybrids and the two commercial checks (SC627 and ZS261) were used to construct  a ‘mean versus stability’ biplot for clear visualization.

Line-by-tester analysis across environments was conducted with SAS for traits that showed significant differences among hybrids using the adjusted means from each environment analysis^46^ excluding checks. The total variations among QPM F_1_ hybrids were partitioned into lines (L), testers (T), and line x tester (L  ×  T) sources of variations. The main effects of inbred lines and testers represent the GCA effects, while L × T interaction represents the SCA effect^47^. The significance of line, tester, and L × T mean squares were tested against the mean squares of their respective interactions with the environment. The mean square attributable to L × T was tested against the mean square for L × T interaction with environment (E), (L × T × E), whereas the mean square for L × T × E was tested using the pooled error mean square. The GCA of inbred lines (GCA_Line_) and testers (GCA_Tester_), as well as the SCA of crosses (SCA) and their respective standard errors, were determined across environments with SAS version 9.3^44^. The statistical model recommended by Arunachalam^48^ and Dabholkar ^49^ was employed for analyzing the performance of L × T across environments:

$${Y}_{ijk}= \mu +{g}_{i}+{g}_{j}+{s}_{ij}+{e}_{k}+({ge)}_{ik}+({ge)}_{jk}+({se)}_{ijk}+ {e}_{ijk}$$

where ${Y}_{ijk}$ is the observed performance of the cross between ith line and jth tester in kth environment, µ is the overall mean, ${g}_{i}$ is GCA effect of the ith line, ${g}_{j}$ is GCA effect of the jth tester, ${S}_{ij}$ is SCA effect of the cross between ith line and jth tester, e_k_ is the environment effect, $({ge)}_{ik}$ is the interaction between GCA effect of the ith line and kth environment,$({ge)}_{jk}$ is the interaction between GCA effect of the jth tester and kth environment, $({se)}_{ijk}$ is the interaction between SCA effects of the cross and environment, and ${e}_{ijk}$ is pooled error for ${Y}_{ijk}$ observation.

GCA and SCA effects and their standard errors were estimated using SAS software version 9.3^44^. The relative importance of GCA (GCA_Line_ + GCA_Tester_) and SCA effects were determined as the proportion of the cross sum of squares to GCA (line and tester sum squares) or SCA^50^. Broad sense heritability (H^2^) across environments was estimated as described by Hallauer et al.^47^ using variance components as:${H}^{2}=\frac{{\updelta }_{{\text{G}}}^{2}}{\left[{\updelta }_{{\text{G}}}^{2}+ \frac{{\updelta }_{{\text{GXE}}}^{2}}{{\text{E}}}+\frac{{\updelta }_{{\text{E}}}^{2}}{{\text{ER}}}\right]}$, where $\delta_{G}^{2}$; $\delta_{GxE}^{2}$ and $\delta_{E}^{2}$ are genotypic, GEI and residual variances, respectively; while E and R represent numbers of environments and replications, respectively.

### Ethics statements

Maize plant data collection was done following the CIMMYT’s guidelines.

## Results and discussion

### Analysis of variance and mean performances

Analysis of variance for each environment showed significant differences among genotypes for grain yield and most agronomic and protein quality traits (Supplementary Table S1). The combined analysis of variance across environments showed significant (*P* ≤ 0.01) environment, genotype (new QPM hybrids and commercial checks), hybrid, and hybrid × environment interaction (HEI) effects for all traits, except HEI effects for protein content and tryptophan concentration and protein quality index (Table 3). Significant differences observed among the genotypes and hybrids for all measured traits across environments demonstrated the existence of adequate genetic variation among the genotypes for the studied traits. The existence of genetic variations among QPM hybrids for grain yield and other agronomic traits under different environments were reported by several investigators^8,30–32,35^. Significant variations among QPM genotypes for kernel endosperm modification, protein and tryptophan concentration were also reported before^29,31,34^. The significant HEI observed in the current study for grain yield and all other agronomic traits justified inconsistent performance of the hybrids across the test environments and the need for testing genotypes across environments to select stable hybrids. Previous studies also showed significant HEI for grain yield and other agronomic traits in QPM hybrids^8,30,31,34,35^. Non-significant HEI for protein and tryptophan contents and protein quality index suggested that the expression of these traits was not affected under different environmental conditions. This indicates that testing genotypes in fewer environments would be adequate to phenotype these traits. In agreement with the current study, non-significant HEI was reported earlier for protein quality traits^29,31^.

**Table 3:** Analysis of variance for grain yield, agronomic traits and protein quality parameters of 106 quality protein maize hybrids and two commercial check hybrids tested across six environments in 2015 and 2016.

	df	GY	AD	DS	df	PH	EH	EPP	df	MOD	PRT	TRP	QI
		t ha^−1^	d	d		cm	cm			(1–5)	g kg^−1^	g kg^−1^	%
Environment (E)	5	97.60**	43,923.73**	40,450.58**	4	189,931.25**	44,915.03**	9.77**	1	60.91**	21,973.99**	4.28**	0.79**
Replication (E)	6	19.91**	19.80**	14.89**	5	1670.93**	557.98**	0.15**	2	1.65**	73.89	0.26**	0.21**
Block (Rep x E)	252	1.75**	5.82**	6.74**	210	222.43**	156.32**	0.04*	84	0.17	61.32	0.08**	0.01**
Genotype	107	6.59**	43.11**	44.97**	107	1039.36**	904.18**	0.12**	107	1.26**	124.31**	0.10**	0.03**
Hybrids	105	4.44**	28.35**	32.18**	105	773.41**	670.86**	0.07**	105	1.25**	101.94**	0.02**	0.03**
GCA_Line_	26	7.64**	90.51**	92.31**	26	1793.92**	1831.26**	0.20**	26	1.39**	185.03**	0.08**	0.09**
GCA_Tester_	3	13.48	99.92**	198.59**	3	5314.89**	3084.87**	0.14	3	23.50**	795.14**	0.08**	0.09**
SCA	76	2.99**	4.26**	5.03**	76	245.02**	178.59**	0.03*	76	0.33	46.16*	0.003	0.004
Genotype x E	535	1.95**	5.48**	6.70**	428	181.13**	143.98**	0.05**	107	0.55**	44.42	0.01	0.000
Hybrids x E	525	1.15**	2.61**	2.62**	420	105.74**	84.17**	0.03**	105	0.48**	42.29	0.01	0.004
GCA_Line_ x E	130	1.53**	4.68**	4.50**	104	144.77**	124.22**	0.04**	26	0.5	65.66	0.01*	0.006
GCA_Tester_ x E	15	7.79**	11.72**	10.03**	12	352.40**	333.41**	0.13**	3	3.46**	89.73	0.01*	0.001
SCA x E	380	0.76	1.55	1.70	304	82.83	60.66	0.02**	76	0.32	31.44	0.004	0.003
Error	630	0.72	1.49	1.54	525	77.82	56.64	0.01	104	0.38	56.60	0.01	0.00
%SS GCA		51.24	89.13	88.67		77.06	80.73	72.36		81.00	67.23	89.40	90.19
%SS SCA		48.76	10.87	11.32		22.93	19.27	27.65		19.00	32.77	10.60	9.80
Mean		6.2	75.4	76.9		243.3	135.0	1.13		2.31	98.7	0.77	0.78
Minimum		2.5	66.5	68.3		211.4	95.5	0.92		1.25	84.1	0.45	0.44
Maximum		7.7	81.6	83.4		271.0	160.7	1.60		3.75	118.1	0.99	1.01
SE (m)		0.35	0.50	0.51		5.15	4.10	0.08		0.44	5.32	0.05	0.04
CV (%)		13.75	1.62	1.61		3.63	5.58	8.88		26.6	7.62	9.11	7.74
Heritability (H^2^)		0.74	0.90	0.89		0.87	0.88	0.61		0.70	0.72	0.87	0.93

AD, Days to anthesis; DS, Days to silking; PH, Plant height; EH, Ear height; EPP, Ears per plant; MOD, Kernel endosperm modification; PRT, Protein content; TRP, Tryptophan concentration; QI, Quality index.

*Significant at the *P* < 0.05 level of probability.

**Significant at the *P* ≤ 0.01 level of probability.

Mean grain yields ranged from 5.25 t ha^–1^ at Chisumbanje to 6.85 t ha^-1^ at Bako. Across environments, grain yield for the entries ranged from 2.47 to 7.66 t ha^-1^ with a mean of 6.18 t ha^-1^ (Supplementary Table S1). The highest-yielding hybrids across environments were H80 (7.66 t ha^-1^), H72 (7.63 t ha^-1^), H78 (7.56 t ha^-1^), H104 (7.42 t ha^-1^) and H16 (7.41 t ha^-1^) (Table 4). These hybrids showed 19–21% and 9–12% grain yield advantage over the QPM (ZS261) and non-QPM (SC627) commercial checks, respectively. Across environments, 28% and 68% of the QPM hybrids had higher grain yield than the commercial non-QPM (SC627) and QPM (ZS261) hybrids, respectively, indicating the genetic progress made in QPM breeding for high yield with enhanced protein quality in ESA. Previous studies also demonstrated the comparative yield advantage of QPM genotypes to conventional maize genotypes adapted to ESA^6,8,30^. Days to anthesis for the hybrids ranged from 67 to 82, with a mean of 75 days, while days to silking varied from 68 to 83 with a mean of 77 days (Table 3, Supplementary Table S1). Among the high-yielding hybrids (Table 4), H11 and H49 mature earlier than all the other hybrids, including the commercial checks. Hybrids with high grain yield and early flowering characteristics are important in areas with short rainy seasons and environments affected by terminal drought. Plant height ranged from 211 to 271 cm, with a mean of 243 cm, and ear height ranged from 96 to 161 cm, with a mean of 135 cm. Hybrids H28, H34 and H11 had shorter plant and ear heights (Table 4). Mean number of ears per plant was 1.12 with a range of 0.92 to 1.60 (Table 3, Supplementary Table S1). Among the selected hybrids, H28, H32, H34, H80 and H104 showed higher number of ears per plant (Table 4).

**Table 4:** Mean performances of the top-yielding 25 quality protein maize testcross hybrids for grain yield, agronomic and protein quality traits evaluated across six environments in 2015 and 2016.

Hybrid	Cross	Grain Yield (t ha^-1^)							Yield advantage (%)	
		BK	AM	GW	GL	MP	CH	AC_LOC	SC627	ZS261
H80	L21 × T2	9.7	6.8	6.7	8.8	8	6.1	7.7	12.99	22.08
H72	L19 × T2	10.2	6.5	8	9.7	7.5	3.8	7.6	11.84	21.05
H78	L20 × T4	6.3	8.5	7.4	9.1	7.7	6.4	7.6	11.84	21.05
H104	L27 × T2	9.4	8.6	7.1	7.6	6.8	5	7.4	9.46	18.92
H16	L4 × T4	6.0	7.6	7.1	10.4	8	5.3	7.4	9.46	18.92
H73	L19 × T3	9.1	6.2	8.4	5.1	8.1	6.6	7.3	8.22	17.81
H28	L7 × T4	5.9	7.1	8.1	8.8	7.3	6	7.2	6.94	16.67
H48	L12 × T4	6.2	5.7	6.9	10.5	7.2	6.4	7.1	5.63	15.49
H60	L15 × T4	6.2	7.7	6.2	9.2	7.6	5.9	7.1	5.63	15.49
H103	L27 × T1	8.0	8.6	7.1	6.1	7.3	5.6	7.1	5.63	15.49
H49	L13 × T1	7.2	7.8	7.0	7.0	6.3	7.2	7.1	5.63	15.49
H76	L20 × T2	7.8	7.5	9.0	5.2	7.5	5.3	7.1	4.29	14.29
H53	L14 × T1	7.9	8.4	6.7	5.7	8.3	5.1	7.0	4.29	14.29
H106	L27 × T4	6.2	6.5	6.9	8.8	7.6	6.0	7.0	4.29	14.29
H50	L13 × T2	7.7	5.5	8.4	8.6	6.8	4.9	7.0	4.29	14.29
H11	L3 × T3	6.8	7.9	7.7	5.5	6.5	7.4	7.0	4.29	14.29
H34	L9 × T2	7.6	6.7	8.9	6.9	7	4.5	6.9	2.90	13.04
H32	L8 × T4	5.6	7	6.4	8.4	7.6	6.8	6.9	2.90	13.04
H56	L14 × T4	6.2	6	7.4	7.1	8.2	6.7	6.9	2.90	13.04
H65	L17 × T1	6.5	6.4	8.1	7.3	6.9	6.3	6.9	2.90	13.04
H75	L20 × T1	7.8	9.2	6.5	5.3	7	5.5	6.9	2.90	13.04
H23	L6 × T3	7.3	6.2	7.2	6.8	7.1	6.6	6.9	2.90	13.04
H79	L21 × T1	8.3	6.4	5.9	6.9	7	6.7	6.9	2.90	13.04
H51	L13 × T3	6.4	6.2	6.9	8.2	7.3	6.2	6.9	2.90	13.04
H22	L6 × T2	9	7.2	6.2	5.9	7	5.8	6.8	1.47	11.76
H107	SC627 (non-QPM)	7.4	7	5.1	6.8	7.2	6.7	6.7		
H108	ZS261 (QPM)	6.5	5.6	5.9	7.1	5.3	7	6		
Mean		6.8	6.2	6.5	5.9	6.5	5.2	6.2	5.58	15.44
LSD		1.8	1.7	1.9	3.5	1.7	1.9	0.9		
NLOC		1	1	1	1	1	1	6		

Hybrid	SCA	AD	DS	PH	EH	EPP	MOD	PRT	TRP	QI
		–-days–		––cm–-		#	5-Jan	–g kg^-1^–		%
H80	0.59*	77	79	250	138	1.3	1.5	105	0.77	0.73
H72	0.5	77	80	258	148	1.2	2	101	0.55	0.55
H78	0.24	76	78	244	139	1.1	3.4	106	0.67	0.63
H104	0.34	78	79	246	132	1.3`	1.6	106	0.82	0.77
H16	1.27**	76	78	245	144	1.2	3.1	94	0.74	0.78
H73	0.5	75	77	261	143	1.2	1.9	96	0.51	0.53
H28	0.79**	74	76	231	121	1.3	3.3	97	0.86	0.88
H48	0.72*	75	78	241	130	1	3.4	95	0.89	0.93
H60	0.45	75	77	239	133	1.1	3	91	0.81	0.88
H103	0.51	75	76	261	148	1.2	2.1	101	0.82	0.81
H49	0.46	73	74	254	142	1.1	2.9	88	0.45	0.51
H76	− 0.17	77	80	255	148	1.1	1.9	112	0.64	0.57
H53	0.58	75	76	242	149	1.1	1.9	96	0.79	0.82
H106	− 0.21	76	78	238	135	1.2	2.4	92	0.85	0.93
H50	− 0.13	75	78	248	135	1.1	2.6	101	0.53	0.53
H11	0.74*	73	74	237	126	1.2	2	95	0.78	0.81
H34	0.45	76	78	233	122	1.3	2	96	0.85	0.88
H32	0.49	76	78	238	126	1.3	2.8	98	0.94	0.95
H56	− 0.09	76	78	246	142	1.1	3.8	106	0.92	0.86
H65	0.91**	73	76	243	140	1.1	2.4	92	0.74	0.8
H75	0.17	76	78	264	154	1.1	3.3	100	0.61	0.61
H23	0.57	74	76	252	142	1	2.6	98	0.78	0.79
H79	0.28	75	76	261	155	1.2	1.8	95	0.69	0.73
H51	0.11	75	76	261	149	1.1	2.3	106	0.46	0.44
H22	0.17	76	78	249	142	1.1	1.9	99	0.74	0.75
H107	–	74	74	255	142	0.9	1	112	0.57	0.48
H108	–	73	73	239	120	1	2	105	0.77	0.72
Mean		75	77	243	135	1.1	2.3	99	0.77	0.78
LSD		1.6	1.7	10.1	8.1	0.1	0.9	10.5	0.1	0.09
NLOC		6	6	5	5	5	2	2	2	2

H107 and H108 are non-QPM and QPM commercial checks, respectively.

*BK* Bako, *AM* Ambo, *GW* Gwebi, *GL* Glendale, *MP* Mpongwe, *CH* Chisumbanje, *AC*_*LOC* Across all locations, *SCA* Specific combining ability (SE ±  = 0.3), *AD* Days to anthesis, *DS* Days to silking, *PH* Plant height, *EH* Ear height, *EPP* Ears per plant, *MOD* Kernel endosperm modification, *PRT* Protein content, *TRP* Tryptophan concentration, *QI* Quality index, *NLOC* Number of locations.

Mean grain protein content was 99 g kg^−1^, with a range of 84–118 g kg^−1^, whereas tryptophan concentration ranged from 0.45 to 0.99 g kg^−1^, with a mean of 0.77 g kg^−1^. Among the top-yielding 25 hybrids, H76 (111 g kg^−1^), H104 (106 g kg^−1^) and H56 (106 g kg^−1^) had higher protein levels, while H32 (0.94 g kg^−1^), H56 (0.92 g kg^−1^) and H48 (0.89 g kg^−1^) had higher grain tryptophan concentrations. These values were higher than the levels of protein (105 g kg^−1^) and tryptophan (0.77 g kg^−1^) concentrations of the commercial QPM check hybrid (ZS261). The mean protein quality index was 0.78%, with a range of 0.44 to 1.01%, whereas endosperm modification ranged from 1.3 to 3.8, with a mean of 2.3. Hybrids H32 (0.95%), H48 (0.93%) and H106 (0.93%) had higher quality index. Hybrids H80 (1.5), H104 (1.6), and H79 (1.8) showed the most desirable level of endosperm modification (Table 4). According to Vivek et al.^37^, standard QPM genotypes should have an endosperm modification score close to 2.0, a quality index of at least 0.80% and 0.75 g kg^−1^ tryptophan concentration, and 80 g kg^−1^ protein content in whole grain. Most QPM hybrids evaluated in this study had above the recommended levels of quality traits than the commercial QPM check hybrid (ZS261), indicating QPM breeding progresses in improving protein quality without grain yield penalty.

Estimated H^2^ across environments was high for grain yield (74%), days to anthesis (90%), days to silking (89%), plant height (87%), ear height (88%), protein content (72%), tryptophan concentration (87%), quality index (93%) and endosperm modification (70%). Moderate H^2^ was recorded for number of ears per plant (61%).

### GGE-biplot analysis

**Figure 1:**
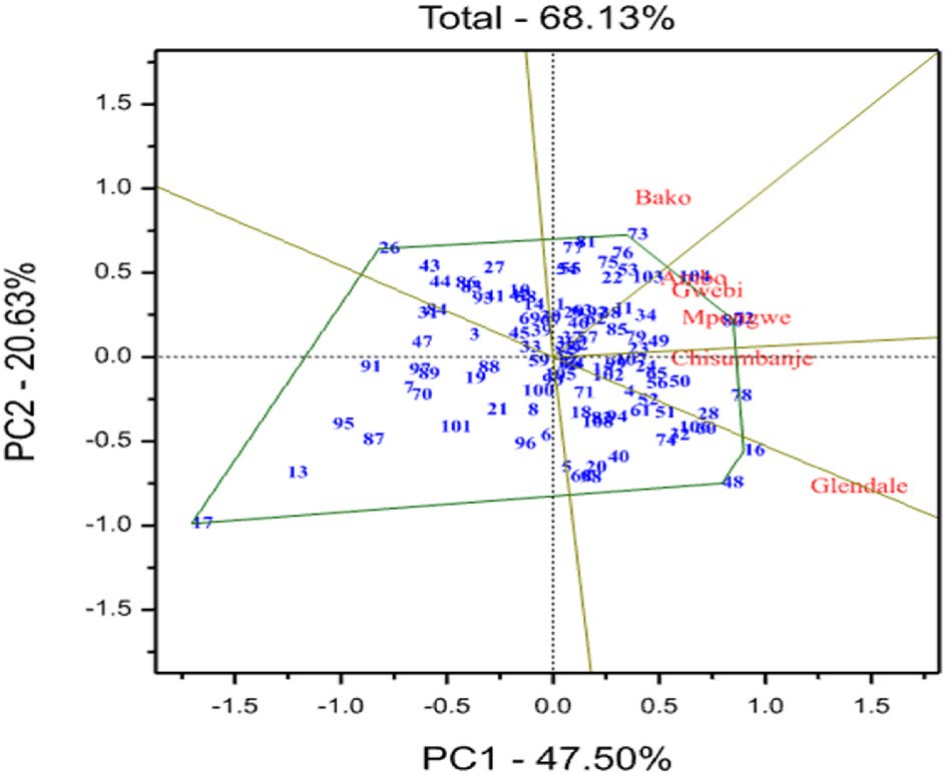
Shows the ‘which-won-where’ view of genotype main effect plus genotype by environment interaction (GGE) biplot constructed based on environment-centered singular-value partitioning for grain yield of 108 genotypes tested across six environments. The codes of genotypes are stated in Supplementary Table S1.

**Figure 2:**
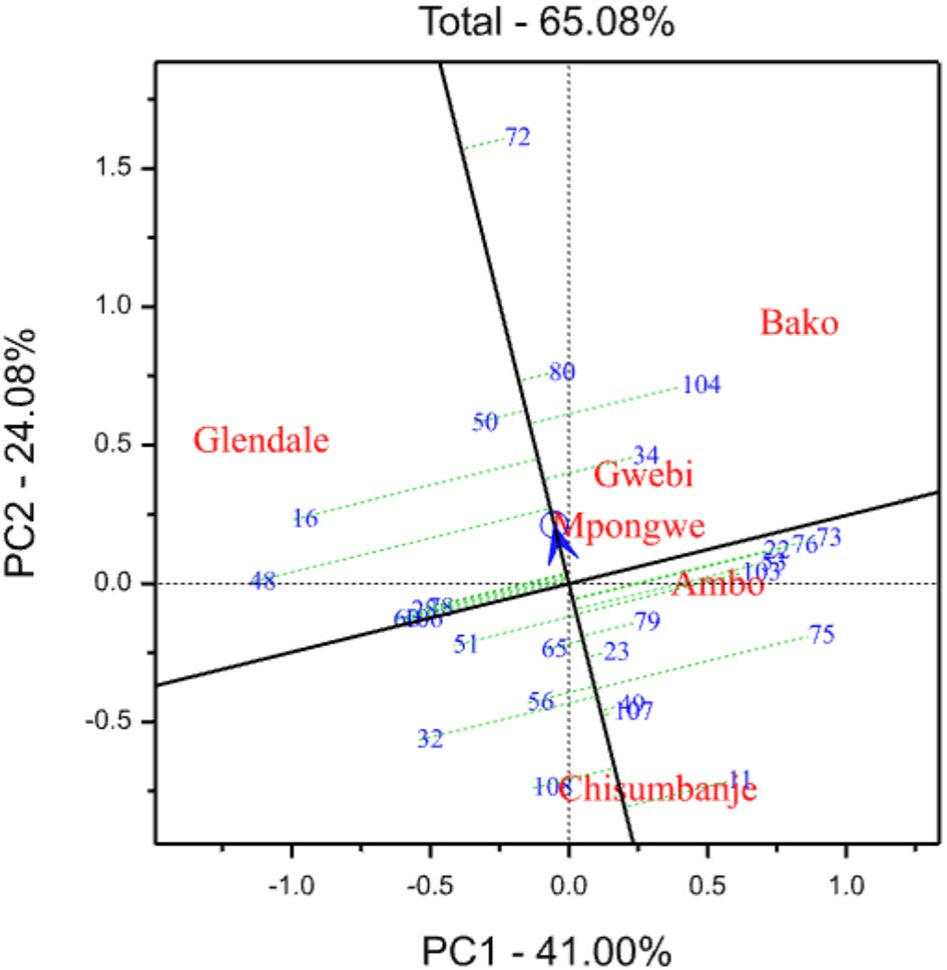
Shows the ‘mean versus stability’ view of the genotype main effect plus genotype by environment interaction (GGE) biplot constructed based on grain yield data of 25 top-yielding quality protein maize hybrids and two commercial checks evaluated across six environments. The codes of genotypes are stated in Supplementary Table S1.

The “which-won-where” pattern of the multi-environment polygon view of Fig. 1 depicted which genotype performed best in which environment (Fig. 1). PC1 and PC2 explained 47.5% and 20.6% of the variation for grain yield, indicating that the biplot accounted for 68.1% of the total variation related to genotype and genotype by environment interaction. The biplot was sub-divided into six sectors and H73, H72, H16, H48, H17 and H26 constituted vertex genotypes in each sector. The winning hybrids were H72, H80 and H104 at Ambo, Gwebi and Mpongwe; H73 at Bako; and H16 and H48 at Glendale. Chisumbanje didn’t discriminate among the hybrids; hence, it was less informative. Two corner hybrids, G17 and G26, were disposed of far from all the test locations on the GGE-biplot, signifying their inferior yield performance at all the environments. The GGE analysis delineated the test environments into two mega-environments (Fig. 1). The first comprised of Ambo, Bako, Chisumbanje, Gwebi and Mpongwe and the second comprised of only a single environment, Glendale. Within the first mega environment Ambo, Chisumbanje, Gwebi and Mpongwe are positively correlated because they are placed in less than 90° in the GGE-biplot. Different authors used AMMI and GGE bi-plot models to know how many mega environments exist in a specific target environment^32,51–53^. Mean and stability of the top 27 hybrids (25 high-yielding testcrosses and two commercial checks) were visualized by drawing an average environment coordination (AEC) view graph, represented by a small circle (Fig. 2). The thick line perpendicular to AEC ordinate separated genotypes with yield less than the average (to the left side line) from those with grain yield greater than the mean (to the right side line). Accordingly, H72, H80, H50, H104, H16, H34 and H48 had higher grain yield across environments. Their projections onto the AEC ordinate measured the stability of the hybrids. Among the highest-yielding hybrids, H50, H80, H72 and H104 were the most stable hybrids that with shorter projections onto the AEC ordinate. High-yielding and stable hybrids identified in this study could be recommended for on-farm testing and commercial production in ESA after fulfilling the requirements for varietal release. However, hybrids like H72 and H50, with very low tryptophan concentration and protein quality index, cannot be advanced further as QPM hybrid. Previous research findings in Southern Africa^6^ and Eastern and Southern Africa^8,34^ also reported stable and high-yielding QPM hybrids.

### Combining ability analysis

Mean squares attributable to line GCA and tester GCA were significant for most traits, except tester GCA for grain yield and ears per plant (Table 3). Similarly, SCA mean squares were significant for most traits, except kernel modification, tryptophan concentration, and quality index, indicating the importance of both additive and non-additive genetic effects in the inheritance of these traits. Thus, effective selection of these traits for further improvement could be feasible through hybridization, recurrent selection and back-cross breeding. The present findings are consistent with previous studies on QPM genotypes^30,32,34,35^. The analysis also revealed highly significant line GCA × environment interactions for most traits, except for kernel modification, protein content, and quality index (Table 3). Significant tester GCA × environment interaction was observed for most traits, except for protein content, tryptophan concentration and quality index. Significant GCA × environment interactions for most measured traits indicate that combining abilities of inbred lines and testers varied across test environments. This implies the need to test the combining abilities of the inbred lines and testers across environments prior to the selection of stable parental genotypes^54^. Significant GCA × environment interaction was reported previously in QPM genotypes across environments for grain yield and agronomic traits^21,31,32,34^. In contrast, Njeri et al.^35^ reported non-significant GCA × E interaction for grain yield across optimally managed environments. This study showed non-significant GCA × environment interaction effects for protein content, tryptophan concentration and protein quality index that would enable selection of QPM inbred lines with stable GCA effects across test environments.

The mean square due to SCA × environment interaction were significant only for ears per plant (Table 3). The existence of non-significant SCA × environment interaction for most studied traits indicated consistent expression of SCA effects for these traits in different environments. Likewise, Machida et al.^29^ and Nepir et al.^31^ reported non-significant SCA interaction with the test environments for agronomic and protein quality traits. Contrary to the current findings, significant SCA × environment interaction was reported for endosperm modification and protein quality traits by Wegary et al.^34^ and Abakemal et al.^55^.

GCA sum of squares (both line and tester GCAs) as a percentage of the hybrid sum of squares were larger than SCA sum of squares for all studied traits. The contribution of GCA sum squares ranged from 51% (grain yield) to 90% (quality index), while the SCA sum of squares ranged from 10 to 49% (Table 3). The greater contribution of the GCA sum of squares among the hybrids for most traits observed in this study implies the preponderance of additive genetic effects for these traits in the set of QPM inbred lines studied. Therefore, progeny performance can adequately be predicted based on parental performances^34,56^. Early generation testing and selecting potential single-cross hybrids through prediction from GCA effects alone could be feasible^54^. The preponderance of additive effect for agronomic and protein quality traits in QPM germplasm have also been reported in previous studies^31,34,35,55^.

Similar contributions of both GCA and SCA (51 vs. 49%) to the hybrid sum of squares for grain yield suggested that both additive and non-additive genetic effects are almost equally important. In such a scenario, breeding programs should exploit both components by evaluating parents for GCA and testing the resulting hybrids in target environments^55,57^. Contrary to the current results, Wegary et al.^34^ reported the importance of non-additive gene effects in the inheritance of grain yield in QPM germplasm.

### Estimates of combining ability effects

The 27 QPM inbred lines and four testers used in this study depicted considerable variations in GCA effects for most studied traits (Table 5), indicating the existence of sizable diversity in the genetic constitution of the inbred lines. For grain yield, L13, L19, L20, L21 and L27 showed highly significant positive GCA effects, indicating that these inbred lines could be useful sources of favorable alleles for higher grain yield. These inbred lines also have greater potential to be used as testers in the breeding program. L1, L2, L3, L9 and L17 showed highly significant negative GCA effects for days to anthesis and silking and for plant and ear height. Such inbred lines could be utilized in early maturing and short-statured QPM hybrid development by considering the yield potential and other desirable attributes. L7, L8 and L16 had highly significant positive GCA effects for number of ears per plant and can be sources of favorable alleles for enhancing prolificacy in QPM germplasm. For kernel endosperm modification, L21, L23 and L24 showed significant negative GCA effects, signifying their value in developing QPM varieties with well-modified kernel endosperm. QPM inbred lines exhibiting modified endosperm phenotype could be used as *o2* donor parents for the conversion of non-QPM inbred lines to QPM counterparts^30,37^. L2, L10, L11 and L20 had desirable GCA effects for protein content, indicating that these inbred lines contain a higher frequency of favorable alleles to elevate protein content in the hybrids. About one-third of the inbred lines studied showed significant and positive GCA effects for tryptophan concentration and protein quality index. L8, L12 and L18 led to these traits’ most desirable GCA effects.

**Table 5:** General combining ability effects (GCA) for grain yield (t ha^−1^), agronomic and protein quality traits for 27 quality protein maize inbred lines and four testers evaluated across environments in 2015 and 2016.

Line	Name	GY	DA	DS	PH	EH	EPP	MOD	PRT	TRP	QI
L1	TL156581	0.09	− 1.37**	− 0.97*	− 8.01**	− 6.74*	0.00	0.03	2.72	0.08**	0.06**
L2	TL156582	− 0.59*	− 7.93**	− 7.88**	− 28.14**	− 31.33**	− 0.14**	− 0.28	8.33**	− 0.15**	− 0.20**
L3	TL148288	0.23	− 2.86**	− 2.66**	− 12.14**	− 8.66**	0.05	0.06	− 3.64	0.01	0.03
L4	TL156585	− 0.32	1.70**	1.28**	− 3.36	8.79**	− 0.04	− 0.31	− 1.10	− 0.07**	− 0.07**
L5	TL156586	− 1.18**	2.31**	2.62**	− 6.24*	− 0.74	− 0.05	0.31	− 8.01**	− 0.02	0.04*
L6	TL156589	0.32	− 0.02	− 0.08	− 0.14	4.72	− 0.10*	0.47*	− 4.62	− 0.01	0.03
L7	TL156590	− 0.05	0.05	− 0.30	− 0.99	− 6.30*	0.20**	− 0.06	0.43	0.08**	0.07**
L8	TL156591	− 0.02	1.83**	1.24**	3.56	− 1.11	0.15**	− 0.22	1.04	0.11**	0.10**
L9	TL148287	0.15	− 1.77**	− 2.09**	− 12.39**	− 12.45**	0.05	0.12	− 2.71	0.08**	0.10**
L10	TL116960	0.19	− 0.62	0.01	2.24	− 7.61**	− 0.05	0.15	6.42**	0.08**	0.02
L11	TL116955	− 0.56*	0.20	− 0.56	19.71**	12.14**	− 0.05	0.12	8.25**	0.11**	0.03
L12	TL13609	− 0.05	0.44	0.44	4.82	− 1.33	− 0.17**	0.31	1.43	0.14**	0.12**
L13	TL156597	0.75**	− 1.03*	− 0.83	5.80*	2.12	− 0.02	0.47*	0.42	− 0.24**	− 0.25**
L14	TL156599	0.56*	0.18	0.07	5.18*	7.16**	− 0.04	− 0.13	3.89	0.06**	0.03**
L15	TL156600	0.20	− 0.21	− 0.38	− 0.69	4.45	− 0.04	− 0.10	− 4.39	0.02	0.05*
L16	TL156604	0.31	0.04	− 0.15	2.09	− 2.95	0.25**	0.09	− 3.34	0.06**	0.09**
L17	TL155932	0.15	− 1.87**	− 1.18*	− 8.52**	− 10.33**	− 0.09*	− 0.35	− 2.21	0.02	0.03
L18	TL155814	− 0.76**	2.20**	2.56**	2.09	3.56	− 0.15**	0.81**	− 4.44	0.14**	0.12**
L19	TL156608	0.77**	0.54	1.29**	13.64**	15.78**	0.07	0.15	2.38	− 0.18**	− 0.20**
L20	TL156609	0.85**	− 0.07	0.45	9.37**	6.80**	− 0.09*	0.69**	7.25**	− 0.13**	− 0.18**
L21	VL05127	0.71**	− 0.10	− 0.29	6.55*	6.64*	0.05	− 0.53*	− 1.66	0.00	0.01
L22	TL135414	− 0.40	3.76**	3.61**	1.24	12.05**	0.09	0.28	2.43	0.02	0.00
L23	TL155933	− 0.97**	2.41**	2.28**	0.10	2.50	0.02	− 0.72**	− 3.99	− 0.01	0.02
L24	TL156601	− 0.29	0.22	0.17	4.74	7.37**	− 0.05	− 0.44*	− 5.04	− 0.02	0.01
L25	TL156605	− 0.95**	3.15**	2.96**	− 11.70**	− 1.73	0.06	− 0.41	− 8.81**	− 0.02	0.06**
L26	VL06375	− 0.24	− 0.11	− 0.03	7.00**	− 4.06	− 0.09*	0.19	− 3.84	0.01	0.05*
L27	TL156611	0.73**	0.49	0.13	5.26*	3.04	0.10*	− 0.28	0.71	0.05*	0.04*
SE (gi)		0.25	0.44	0.47	2.64	2.45	0.04	0.22	2.66	0.02	0.02
Tester											
T1	TL156587	− 0.30	0.21	− 0.12	0.53	5.53**	0.01	0.14	− 3.96**	− 0.04**	− 0.02*
T2	VL05552	0.18	1.00**	1.42**	− 0.02	− 3.34**	0.05	− 0.58**	4.89**	0.01	− 0.03**
T3	TL148289	− 0.19	− 0.74**	− 1.29**	7.42**	2.51	− 0.03	− 0.35**	− 0.29	0.01	0.01
T4	TL149662	0.30	− 0.42	0.05	− 7.92**	− 4.62**	− 0.02	0.78**	− 2.70**	0.04**	0.07**
SE (gj)		0.19	0.22	0.27	1.40	1.36	0.03	0.08	1.02	0.01	0.01

GY, Grain yield; AD, Days to anthesis; DS, Days to silking; PH, Plant height; EH, Ear height; EPP, Ears per plant; MOD, Kernel endosperm modification; PRT, Protein concentration; TRP, Tryptophan concentration; QI, Quality index; SE(gi), Standard error of GCA effects for line (L); SE (gj), Standard error of GCA effects for tester (T).

*Significant at the *P* ≤ 0.05 probability level.

**Significant at the *P* ≤ 0.01 probability level.

None of the testers showed significant GCA effects for grain yield (Table 5). However, this study identified testers with desirable GCA effects and, hence, favorable additive effects for days to anthesis and silking (T3), plant and ear height (T4), endosperm modification (T2 and T3), protein content (T2), tryptophan concentration and protein quality index (T4). The presence of significant and desirable inbred line and tester GCA effects for most of the studied traits indicated the breeding value of the parents attributable to additive genetic effects that enable breeders to predict progeny performance based on  parental performances. Highly variable SCA effects that ranged from negative to positive were observed among the line-by-tester cross combinations for grain yield, days to anthesis and silking, plant and ear height, ears per plant, and protein content (Supplementary Table S2). This indicated that specific crosses performed better or poorer than what could be expected based on the GCA effects of respective parental inbred lines and/or testers. This can be witnessed by the fact that none of the cross combinations with best SCA effects for grain yield, which were also among the high-yielding hybrids across environments, viz. L4 × T4 (H16), L7 × T4 (H28) and L17 × T1 (H65), contain parents with high GCA effects for the same trait.

## Conclusions

The current study's results revealed a high level of genetic variations among the QPM hybrids for yield-related and protein-quality traits, hence the possibility of genetic improvement through selection. Hybrids H80 and H104, with stable performance and improved protein quality, were identified for further testing and commercialization. The grain yield level and protein quality improvement observed in this study proved the genetic progresse made in CIMMYT’s QPM breeding program that generated QPM germplasm as good as or even better than the non-QPM. The predominance of additive genetic effect for nearly all the traits observed in this study may reflect the desirable gene flow from parents and fewer environmental effects that guarantee the selection of superior QPM varieties adapted to ESA. The present study identified several QPM inbred lines with highly desirable GCA effects that can be sources of favorable alleles for developing desirable QPM hybrids and synthetics.

### Supplementary Information

Supplementary Table 1.

Supplementary Table 2.

Supplementary Legends.

## Data Availability

All relevant data are included in this article and its supplementary files. Raw data that support  the findings of this study’s are available from the corresponding author upon request.

## Abbreviations

AEC: Average environment coordination
CIMMYT: International Maize and Wheat Improvement Center
DA: Days to anthesis
DS: Days to silking
EH: Ear height
EPP: Ears per plant
ESA: Eastern and Southern Africa
GCA: General combining ability
GEI: Genotype by environment interaction
GGE: Genotype main effect plus genotype by environment interaction
GY: Grain yield
HEI: Hybrid by environment interaction
L: Line
L × T: Line by tester
MOD: Endosperm modification
NARS: National Agricultural Research Systems
PC: Principal component
PH: Plant height
PRT: Protein content
QI: Quality index
QPM: Quality protein maize
SCA: Specific combining ability
SSA: Sub-Sahara Africa
SVD: Singular value decomposition
T: Tester
TRP: Tryptophan concentration
