# Supplementary material for: Testcross performance and combining ability of early-medium maturing quality protein maize inbred lines in Eastern and Southern Africa
Source: Sci Rep. 2024 Apr 21;14:9151. doi: 10.1038/s41598-024-58816-y (PMC11033265; doi:10.1038/s41598-024-58816-y)
Supplement: Supplementary file 1 — Supplementary Table 1. [file 41598_2024_58816_MOESM1_ESM.docx]

**Supplementary Table S1** Mean performances of 106 testcross hybrids, commercial and local checks for grain yield, agronomic and protein quality traits evaluated across six locations in Southern and Eastern Africa during the 2015 and 2016 cropping seasons

| Hybrid | Name | Cross | Grain Yield (t ha^-1^) | | | | | | | AD | DS | PH | EH | | EPP | | MOD | PRT | | TRP | QI |
| --- | --- | --- | --- | --- | --- | --- | --- | --- | --- | --- | --- | --- | --- | --- | --- | --- | --- | --- | --- | --- | --- |
|  |  |  | BK | AM | GW | GL | MP | CH | AC_LOC | ---days-- | | ----cm--- | | --#- | | 1-5 | | | ---g kg^-1^--- | | % |
| H1 | TH15855 | L1 x T1 | 7.49 | 7.01 | 6.84 | 5.12 | 6.06 | 5.90 | 6.40 | 74 | 76 | 236 | 136 | | 1.05 | | 2.50 | 105 | | 0.85 | 0.80 |
| H2 | TH15857 | L1 x T2 | 7.19 | 6.09 | 6.50 | 5.96 | 6.94 | 6.01 | 6.45 | 75 | 78 | 236 | 125 | | 1.20 | | 1.75 | 110 | | 0.90 | 0.82 |
| H3 | TH15859 | L1 x T3 | 7.21 | 6.69 | 5.55 | 4.69 | 4.29 | 4.66 | 5.51 | 74 | 75 | 241 | 130 | | 1.05 | | 1.88 | 97 | | 0.78 | 0.80 |
| H4 | TH15860 | L1 x T4 | 5.85 | 8.13 | 6.35 | 7.34 | 6.77 | 5.60 | 6.67 | 73 | 75 | 228 | 122 | | 1.20 | | 3.25 | 94 | | 0.88 | 0.93 |
| H5 | TH15861 | L2 x T1 | 4.97 | 7.28 | 5.52 | 7.65 | 4.10 | 5.74 | 5.88 | 68 | 70 | 220 | 113 | | 0.96 | | 2.25 | 118 | | 0.54 | 0.46 |
| H6 | TH112116 | L2 x T2 | 5.45 | 6.40 | 5.67 | 6.73 | 4.93 | 5.76 | 5.82 | 68 | 70 | 215 | 96 | | 1.04 | | 1.50 | 115 | | 0.67 | 0.58 |
| H7 | TH15864 | L2 x T3 | 5.66 | 6.28 | 4.84 | 4.17 | 3.56 | 4.26 | 4.79 | 67 | 68 | 215 | 101 | | 0.99 | | 1.75 | 104 | | 0.66 | 0.63 |
| H8 | TH15865 | L2 x T4 | 4.54 | 6.32 | 7.09 | 5.55 | 5.92 | 5.65 | 5.85 | 67 | 69 | 211 | 105 | | 0.94 | | 2.63 | 96 | | 0.59 | 0.62 |
| H9 | TH15872 | L3 x T1 | 6.14 | 7.83 | 6.24 | 5.71 | 6.75 | 5.69 | 6.39 | 73 | 75 | 226 | 128 | | 1.19 | | 2.88 | 85 | | 0.68 | 0.79 |
| H10 | TH15874 | L3 x T2 | 7.82 | 5.34 | 7.62 | 4.36 | 6.06 | 4.88 | 6.01 | 71 | 74 | 238 | 131 | | 1.12 | | 1.63 | 107 | | 0.82 | 0.76 |
| H11 | TH15876 | L3 x T3 | 6.77 | 7.89 | 7.71 | 5.49 | 6.53 | 7.39 | 6.96 | 73 | 74 | 237 | 126 | | 1.17 | | 2.00 | 95 | | 0.78 | 0.81 |
| H12 | TH15877 | L3 x T4 | 6.86 | 5.18 | 6.93 | 5.62 | 6.04 | 6.89 | 6.25 | 73 | 74 | 224 | 121 | | 1.23 | | 3.00 | 92 | | 0.81 | 0.87 |
| H13 | TH15884 | L4 x T1 | 4.18 | 2.57 | 3.50 | 3.23 | 4.39 | 3.13 | 3.50 | 79 | 80 | 221 | 135 | | 0.93 | | 2.00 | 93 | | 0.61 | 0.65 |
| H14 | TH15886 | L4 x T2 | 7.50 | 6.21 | 7.39 | 4.92 | 5.93 | 4.43 | 6.06 | 77 | 79 | 238 | 142 | | 1.15 | | 1.38 | 109 | | 0.73 | 0.66 |
| H15 | TH15888 | L4 x T3 | 7.67 | 6.30 | 5.85 | 7.14 | 6.27 | 5.28 | 6.42 | 76 | 76 | 255 | 154 | | 1.09 | | 1.50 | 94 | | 0.71 | 0.74 |
| H16 | TH15889 | L4 x T4 | 6.02 | 7.59 | 7.14 | 10.40 | 8.01 | 5.32 | 7.41 | 76 | 78 | 245 | 144 | | 1.17 | | 3.13 | 94 | | 0.74 | 0.78 |
| H17 | TH15890 | L5 x T1 | 3.23 | 2.89 | 1.92 | 2.93 | 2.22 | 1.60 | 2.47 | 79 | 80 | 222 | 126 | | 1.16 | | 2.50 | 88 | | 0.70 | 0.79 |
| H18 | TH15892 | L5 x T2 | 7.16 | 4.37 | 6.15 | 7.36 | 6.40 | 4.95 | 6.06 | 79 | 82 | 237 | 137 | | 1.14 | | 2.25 | 98 | | 0.79 | 0.80 |
| H19 | TH15894 | L5 x T3 | 5.65 | 4.47 | 6.90 | 4.51 | 6.31 | 4.26 | 5.35 | 76 | 77 | 254 | 141 | | 1.00 | | 2.38 | 89 | | 0.75 | 0.85 |
| H20 | TH15895 | L5 x T4 | 4.79 | 5.40 | 6.02 | 7.55 | 7.40 | 5.34 | 6.08 | 77 | 79 | 235 | 133 | | 1.02 | | 3.38 | 90 | | 0.77 | 0.85 |
| H21 | TH15905 | L6 x T1 | 5.95 | 5.83 | 5.77 | 5.81 | 4.25 | 4.89 | 5.42 | 76 | 77 | 235 | 136 | | 0.99 | | 3.13 | 92 | | 0.74 | 0.79 |
| H22 | TH15907 | L6 x T2 | 9.02 | 7.19 | 6.16 | 5.93 | 6.98 | 5.76 | 6.84 | 76 | 78 | 249 | 142 | | 1.06 | | 1.88 | 99 | | 0.74 | 0.75 |
| H23 | TH15909 | L6 x T3 | 7.35 | 6.23 | 7.15 | 6.76 | 7.10 | 6.63 | 6.87 | 74 | 76 | 252 | 142 | | 1.03 | | 2.63 | 98 | | 0.78 | 0.79 |
| H24 | TH15910 | L6 x T4 | 6.28 | 7.29 | 7.59 | 7.17 | 7.46 | 5.19 | 6.83 | 75 | 77 | 237 | 139 | | 1.03 | | 3.50 | 89 | | 0.80 | 0.89 |
| H25 | TH15911 | L7 x T1 | 6.95 | 6.13 | 6.91 | 5.83 | 6.28 | 5.21 | 6.22 | 75 | 77 | 245 | 136 | | 1.29 | | 2.38 | 100 | | 0.81 | 0.80 |
| H26 | TH15912 | L7 x T2 | 8.23 | 5.30 | 5.64 | 1.95 | 5.45 | 3.65 | 5.04 | 77 | 79 | 235 | 121 | | 1.42 | | 2.00 | 100 | | 0.80 | 0.79 |
| H27 | TH15914 | L7 x T3 | 6.89 | 5.65 | 7.07 | 2.53 | 7.25 | 6.75 | 6.02 | 75 | 75 | 259 | 136 | | 1.34 | | 1.38 | 101 | | 0.92 | 0.91 |
| H28 | TH15915 | L7 x T4 | 5.91 | 7.11 | 8.11 | 8.77 | 7.32 | 6.00 | 7.20 | 74 | 76 | 231 | 121 | | 1.26 | | 3.25 | 97 | | 0.86 | 0.88 |
| H29 | TH15916 | L8 x T1 | 7.53 | 7.14 | 7.11 | 5.81 | 6.24 | 4.31 | 6.36 | 76 | 78 | 245 | 144 | | 1.32 | | 2.13 | 97 | | 0.78 | 0.81 |
| H30 | TH15918 | L8 x T2 | 7.63 | 6.55 | 6.49 | 5.47 | 6.38 | 4.40 | 6.15 | 81 | 80 | 245 | 128 | | 1.33 | | 1.75 | 102 | | 0.90 | 0.88 |
| H31 | TH15920 | L8 x T3 | 6.81 | 7.11 | 4.63 | 3.38 | 5.20 | 3.83 | 5.16 | 76 | 76 | 259 | 137 | | 1.20 | | 1.75 | 103 | | 0.89 | 0.86 |
| H32 | TH15921 | L8 x T4 | 5.60 | 6.98 | 6.38 | 8.35 | 7.55 | 6.78 | 6.94 | 76 | 78 | 238 | 126 | | 1.27 | | 2.75 | 98 | | 0.94 | 0.95 |
| H33 | TH15927 | L9 x T1 | 7.08 | 4.83 | 7.04 | 5.18 | 6.07 | 5.39 | 5.93 | 74 | 74 | 233 | 129 | | 1.00 | | 2.25 | 95 | | 0.76 | 0.79 |
| H34 | TH15929 | L9 x T2 | 7.58 | 6.75 | 8.93 | 6.89 | 7.01 | 4.53 | 6.95 | 76 | 78 | 233 | 122 | | 1.32 | | 2.00 | 96 | | 0.85 | 0.88 |
| H35 | TH15931 | L9 x T3 | 6.95 | 6.75 | 6.00 | 5.82 | 5.68 | 6.09 | 6.22 | 72 | 73 | 240 | 128 | | 1.17 | | 2.25 | 95 | | 0.85 | 0.89 |
| H36 | TH15932 | L9 x T4 | 7.94 | 4.91 | 7.39 | 6.21 | 5.82 | 4.79 | 6.18 | 73 | 75 | 218 | 112 | | 1.22 | | 3.25 | 99 | | 0.93 | 0.95 |
| H37 | TH15933 | L10 x T1 | 7.17 | 6.37 | 7.29 | 5.89 | 5.83 | 5.20 | 6.29 | 75 | 77 | 250 | 133 | | 1.02 | | 3.25 | 100 | | 0.80 | 0.79 |
| H38 | TH15935 | L10 x T2 | 7.76 | 7.21 | 6.31 | 6.00 | 7.20 | 5.94 | 6.74 | 76 | 79 | 251 | 127 | | 1.16 | | 1.38 | 109 | | 0.81 | 0.75 |
| H39 | TH15937 | L10 x T3 | 6.91 | 6.43 | 6.35 | 4.93 | 6.14 | 5.97 | 6.12 | 73 | 75 | 249 | 128 | | 1.10 | | 1.75 | 108 | | 0.90 | 0.83 |
| H40 | TH15938 | L10 x T4 | 5.45 | 4.98 | 7.02 | 7.97 | 6.71 | 5.63 | 6.29 | 75 | 78 | 232 | 121 | | 1.01 | | 3.50 | 105 | | 0.89 | 0.84 |
|  |  |  |  |  |  |  |  |  |  |  |  |  |  | |  | |  |  | |  |  |
|  |  |  |  |  |  |  |  |  |  |  |  |  |  | |  | |  |  | |  |  |

**Supplementary Table 1** Continued

| Hybrid | Name | Cross | Grain Yield (t ha^-1^) | | | | | | | AD | DS | PH | EH | | EPP | | MOD | PRT | | TRP | QI |
| --- | --- | --- | --- | --- | --- | --- | --- | --- | --- | --- | --- | --- | --- | --- | --- | --- | --- | --- | --- | --- | --- |
|  |  |  | BK | AM | GW | GL | MP | CH | AC_LOC | ---days-- | | ----cm--- | | --#- | | 1-5 | | | ---g kg^-1^--- | | % |
| H41 | TH15972 | L11 x T1 | 8.23 | 7.53 | 4.86 | 4.77 | 5.13 | 3.82 | 5.72 | 76 | 75 | 266 | 158 | | 1.16 | | 2.88 | 103 | | 0.84 | 0.82 |
| H42 | TH113584 | L11 x T2 | 7.84 | 4.67 | 7.36 | 4.32 | 6.96 | 4.84 | 6.00 | 76 | 77 | 265 | 143 | | 0.97 | | 1.63 | 117 | | 0.88 | 0.75 |
| H43 | TH15975 | L11 x T3 | 8.51 | 7.04 | 4.29 | 3.17 | 4.73 | 4.12 | 5.31 | 76 | 76 | 271 | 151 | | 1.15 | | 1.88 | 103 | | 0.84 | 0.81 |
| H44 | TH15976 | L11 x T4 | 7.13 | 7.50 | 5.25 | 2.89 | 4.78 | 4.97 | 5.42 | 75 | 76 | 250 | 137 | | 1.04 | | 3.38 | 107 | | 0.94 | 0.88 |
| H45 | TH15989 | L12 x T1 | 6.31 | 6.79 | 6.11 | 4.40 | 6.13 | 5.95 | 5.95 | 76 | 76 | 250 | 135 | | 0.92 | | 3.13 | 102 | | 0.87 | 0.85 |
| H46 | TH15991 | L12 x T2 | 7.74 | 5.36 | 7.30 | 5.75 | 7.06 | 5.12 | 6.39 | 76 | 78 | 242 | 126 | | 0.99 | | 2.00 | 105 | | 0.99 | 0.94 |
| H47 | TH15993 | L12 x T3 | 6.51 | 5.94 | 4.66 | 3.57 | 5.35 | 4.05 | 5.01 | 76 | 78 | 259 | 143 | | 0.92 | | 2.00 | 99 | | 0.87 | 0.87 |
| H48 | TH15994 | L12 x T4 | 6.21 | 5.71 | 6.95 | 10.48 | 7.16 | 6.35 | 7.14 | 75 | 78 | 241 | 130 | | 1.00 | | 3.38 | 95 | | 0.89 | 0.93 |
| H49 | TH15996 | L13 x T1 | 7.23 | 7.83 | 7.00 | 6.95 | 6.32 | 7.23 | 7.09 | 73 | 74 | 254 | 142 | | 1.14 | | 2.88 | 88 | | 0.45 | 0.51 |
| H50 | TH15998 | L13 x T2 | 7.65 | 5.54 | 8.37 | 8.61 | 6.81 | 4.92 | 6.98 | 75 | 78 | 248 | 135 | | 1.13 | | 2.63 | 101 | | 0.53 | 0.53 |
| H51 | TH151000 | L13 x T3 | 6.45 | 6.17 | 6.87 | 8.17 | 7.28 | 6.17 | 6.85 | 75 | 77 | 261 | 149 | | 1.07 | | 2.25 | 106 | | 0.46 | 0.44 |
| H52 | TH151001 | L13 x T4 | 6.50 | 6.63 | 5.85 | 7.55 | 7.57 | 6.59 | 6.78 | 74 | 76 | 233 | 123 | | 1.08 | | 3.38 | 103 | | 0.66 | 0.65 |
| H53 | TH151012 | L14 x T1 | 7.94 | 8.41 | 6.67 | 5.68 | 8.28 | 5.10 | 7.01 | 75 | 76 | 242 | 149 | | 1.14 | | 1.88 | 96 | | 0.79 | 0.82 |
| H54 | TH151014 | L14 x T2 | 8.06 | 5.83 | 7.96 | 4.61 | 7.31 | 5.23 | 6.50 | 77 | 79 | 250 | 135 | | 1.06 | | 1.63 | 107 | | 0.84 | 0.78 |
| H55 | TH151016 | L14 x T3 | 8.33 | 6.62 | 7.51 | 5.08 | 6.99 | 4.36 | 6.48 | 75 | 76 | 256 | 142 | | 1.07 | | 1.50 | 104 | | 0.74 | 0.71 |
| H56 | TH151017 | L14 x T4 | 6.22 | 6.02 | 7.38 | 7.09 | 8.18 | 6.74 | 6.94 | 76 | 78 | 246 | 142 | | 1.09 | | 3.75 | 106 | | 0.92 | 0.86 |
| H57 | TH151018 | L15 x T1 | 7.72 | 7.04 | 5.87 | 6.35 | 6.20 | 5.33 | 6.42 | 75 | 76 | 241 | 148 | | 1.18 | | 2.25 | 90 | | 0.69 | 0.76 |
| H58 | TH151020 | L15 x T2 | 7.68 | 4.47 | 7.70 | 4.31 | 7.17 | 5.01 | 6.06 | 76 | 77 | 245 | 132 | | 1.09 | | 1.50 | 99 | | 0.82 | 0.83 |
| H59 | TH151022 | L15 x T3 | 7.30 | 5.55 | 5.68 | 5.92 | 6.51 | 4.43 | 5.90 | 74 | 75 | 246 | 144 | | 0.97 | | 2.13 | 99 | | 0.83 | 0.84 |
| H60 | TH151023 | L15 x T4 | 6.17 | 7.67 | 6.23 | 9.17 | 7.61 | 5.85 | 7.12 | 75 | 77 | 239 | 133 | | 1.10 | | 3.00 | 91 | | 0.81 | 0.88 |
| H61 | TH151060 | L16 x T1 | 6.60 | 5.52 | 7.00 | 8.02 | 7.42 | 4.99 | 6.59 | 75 | 76 | 254 | 142 | | 1.40 | | 2.88 | 89 | | 0.79 | 0.89 |
| H62 | TH151062 | L16 x T2 | 7.70 | 5.05 | 7.90 | 5.76 | 7.45 | 5.59 | 6.58 | 77 | 78 | 247 | 127 | | 1.60 | | 1.63 | 98 | | 0.80 | 0.82 |
| H63 | TH151064 | L16 x T3 | 7.89 | 5.12 | 7.70 | 5.35 | 6.72 | 6.34 | 6.52 | 74 | 75 | 248 | 133 | | 1.23 | | 2.00 | 98 | | 0.83 | 0.84 |
| H64 | TH151065 | L16 x T4 | 6.85 | 4.85 | 7.45 | 6.01 | 6.77 | 5.48 | 6.24 | 75 | 77 | 232 | 125 | | 1.26 | | 3.13 | 98 | | 0.93 | 0.93 |
| H65 | TH151078 | L17 x T1 | 6.51 | 6.42 | 8.12 | 7.30 | 6.91 | 6.32 | 6.93 | 73 | 76 | 243 | 140 | | 1.14 | | 2.38 | 92 | | 0.74 | 0.80 |
| H66 | TH132574 | L17 x T2 | 6.28 | 6.37 | 7.13 | 6.01 | 6.59 | 4.75 | 6.19 | 73 | 76 | 229 | 111 | | 1.02 | | 1.38 | 96 | | 0.73 | 0.75 |
| H67 | TH151081 | L17 x T3 | 7.02 | 6.08 | 7.17 | 4.90 | 6.39 | 5.80 | 6.23 | 73 | 74 | 244 | 127 | | 0.98 | | 1.88 | 102 | | 0.90 | 0.88 |
| H68 | TH151082 | L17 x T4 | 4.98 | 5.05 | 5.97 | 7.53 | 6.07 | 6.04 | 5.94 | 74 | 77 | 224 | 120 | | 1.03 | | 2.25 | 99 | | 0.82 | 0.82 |
| H69 | TH151127 | L18 x T3 | 8.23 | 6.18 | 5.75 | 5.36 | 5.30 | 5.05 | 5.98 | 77 | 79 | 261 | 146 | | 0.99 | | 2.63 | 96 | | 0.88 | 0.92 |
| H70 | TH151128 | L18 x T4 | 5.25 | 5.16 | 5.01 | 4.00 | 5.68 | 3.94 | 4.84 | 78 | 80 | 230 | 131 | | 0.97 | | 3.63 | 93 | | 0.94 | 1.01 |
| H71 | TH151135 | L19 x T1 | 7.07 | 5.89 | 4.40 | 6.73 | 7.40 | 5.93 | 6.24 | 76 | 78 | 264 | 161 | | 1.33 | | 2.88 | 103 | | 0.71 | 0.69 |
| H72 | TH151137 | L19 x T2 | 10.18 | 6.55 | 7.99 | 9.75 | 7.51 | 3.78 | 7.63 | 77 | 80 | 258 | 148 | | 1.19 | | 2.00 | 101 | | 0.55 | 0.55 |
| H73 | TH151139 | L19 x T3 | 9.13 | 6.22 | 8.41 | 5.13 | 8.07 | 6.61 | 7.26 | 75 | 77 | 261 | 143 | | 1.21 | | 1.88 | 96 | | 0.51 | 0.53 |
| H74 | TH151140 | L19 x T4 | 6.45 | 6.51 | 6.97 | 9.27 | 6.22 | 4.54 | 6.66 | 76 | 78 | 245 | 152 | | 1.05 | | 3.13 | 107 | | 0.64 | 0.59 |
| H75 | TH151141 | L20 x T1 | 7.81 | 9.23 | 6.54 | 5.26 | 7.02 | 5.49 | 6.89 | 76 | 78 | 264 | 154 | | 1.07 | | 3.25 | 100 | | 0.61 | 0.61 |
| H76 | TH151142 | L20 x T2 | 7.79 | 7.51 | 8.97 | 5.18 | 7.45 | 5.33 | 7.04 | 77 | 80 | 255 | 148 | | 1.05 | | 1.88 | 112 | | 0.64 | 0.57 |
| H77 | TH151143 | L20 x T3 | 8.03 | 7.98 | 7.90 | 4.69 | 6.34 | 4.69 | 6.60 | 72 | 74 | 248 | 126 | | 0.97 | | 3.50 | 109 | | 0.63 | 0.58 |
| H78 | TH151144 | L20 x T4 | 6.32 | 8.46 | 7.39 | 9.07 | 7.72 | 6.40 | 7.56 | 76 | 78 | 244 | 139 | | 1.07 | | 3.38 | 106 | | 0.67 | 0.63 |
| H79 | TH151145 | L21 x T1 | 8.34 | 6.40 | 5.86 | 6.90 | 6.97 | 6.74 | 6.87 | 75 | 76 | 261 | 155 | | 1.24 | | 1.75 | 95 | | 0.69 | 0.73 |
| H80 | TH151147 | L21 x T2 | 9.65 | 6.79 | 6.67 | 8.78 | 7.98 | 6.10 | 7.66 | 77 | 79 | 250 | 138 | | 1.28 | | 1.50 | 105 | | 0.77 | 0.73 |

**Supplementary Table 1** Continued

| Hybrid | Name | Cross | Grain Yield (t ha^-1^) | | | | | | | AD | DS | PH | EH | | EPP | | MOD | PRT | | TRP | QI |
| --- | --- | --- | --- | --- | --- | --- | --- | --- | --- | --- | --- | --- | --- | --- | --- | --- | --- | --- | --- | --- | --- |
|  |  |  | BK | AM | GW | GL | MP | CH | AC_LOC | ---days-- | | ----cm--- | | --#- | | 1-5 | | | ---g kg^-1^--- | | % |
| H81 | TH151149 | L21 x T3 | 8.64 | 8.03 | 6.75 | 5.09 | 7.82 | 3.80 | 6.69 | 74 | 75 | 249 | 139 | | 1.10 | | 1.50 | 97 | | 0.83 | 0.85 |
| H82 | TH151150 | L21 x T4 | 5.25 | 6.54 | 6.54 | 6.92 | 6.98 | 5.69 | 6.32 | 75 | 76 | 239 | 135 | | 1.09 | | 2.38 | 93 | | 0.77 | 0.83 |
| H83 | TH151083 | L22 x T1 | 7.85 | 6.78 | 5.84 | 4.01 | 5.26 | 3.70 | 5.57 | 78 | 80 | 252 | 153 | | 1.14 | | 2.88 | 100 | | 0.76 | 0.76 |
| H84 | TH151084 | L22 x T2 | 7.52 | 4.53 | 4.98 | 3.29 | 7.05 | 4.07 | 5.24 | 82 | 83 | 234 | 140 | | 1.19 | | 1.50 | 107 | | 0.83 | 0.77 |
| H85 | TH151086 | L22 x T3 | 8.61 | 5.85 | 6.29 | 6.80 | 6.88 | 5.64 | 6.68 | 79 | 80 | 254 | 150 | | 1.30 | | 2.38 | 100 | | 0.77 | 0.77 |
| H86 | TH151087 | L22 x T4 | 6.23 | 6.27 | 7.70 | 2.97 | 6.86 | 3.58 | 5.60 | 78 | 80 | 238 | 145 | | 1.22 | | 3.63 | 100 | | 0.83 | 0.83 |
| H87 | TH15849 | L23 x T1 | 3.76 | 3.77 | 5.31 | 3.41 | 5.86 | 3.74 | 4.31 | 81 | 82 | 234 | 137 | | 1.12 | | 1.25 | 89 | | 0.68 | 0.76 |
| H88 | TH15851 | L23 x T2 | 5.95 | 5.82 | 6.28 | 4.95 | 6.30 | 3.44 | 5.46 | 78 | 79 | 246 | 138 | | 1.17 | | 1.25 | 106 | | 0.78 | 0.74 |
| H89 | TH15853 | L23 x T3 | 5.34 | 5.19 | 5.43 | 3.69 | 6.41 | 3.83 | 4.98 | 77 | 78 | 253 | 142 | | 1.12 | | 1.38 | 94 | | 0.80 | 0.84 |
| H90 | TH15854 | L23 x T4 | 5.13 | 6.06 | 7.22 | 5.27 | 7.00 | 5.75 | 6.07 | 76 | 78 | 240 | 133 | | 1.17 | | 2.50 | 91 | | 0.78 | 0.85 |
| H91 | TH151034 | L24 x T1 | 5.20 | 4.97 | 5.77 | 2.59 | 4.13 | 4.71 | 4.56 | 76 | 77 | 244 | 144 | | 0.97 | | 1.88 | 85 | | 0.65 | 0.78 |
| H92 | TH151036 | L24 x T2 | 6.84 | 5.95 | 8.85 | 5.05 | 6.15 | 7.48 | 6.72 | 77 | 79 | 253 | 140 | | 1.25 | | 1.50 | 98 | | 0.77 | 0.78 |
| H93 | TH151038 | L24 x T3 | 5.31 | 6.15 | 7.15 | 2.41 | 7.93 | 6.08 | 5.84 | 74 | 74 | 253 | 145 | | 1.07 | | 1.75 | 103 | | 0.77 | 0.74 |
| H94 | TH151039 | L24 x T4 | 6.10 | 5.48 | 7.23 | 7.43 | 6.41 | 5.91 | 6.43 | 76 | 78 | 242 | 141 | | 1.03 | | 2.38 | 88 | | 0.77 | 0.87 |
| H95 | TH151066 | L25 x T1 | 3.92 | 3.94 | 5.39 | 2.99 | 5.39 | 2.76 | 4.06 | 79 | 80 | 219 | 132 | | 1.23 | | 1.50 | 86 | | 0.72 | 0.84 |
| H96 | TH151068 | L25 x T2 | 5.71 | 4.82 | 5.56 | 6.68 | 5.84 | 4.45 | 5.51 | 79 | 81 | 234 | 136 | | 1.20 | | 1.75 | 100 | | 0.73 | 0.73 |
| H97 | TH151070 | L25 x T3 | 5.37 | 4.71 | 6.35 | 3.59 | 5.91 | 3.45 | 4.90 | 79 | 80 | 241 | 138 | | 1.13 | | 1.50 | 84 | | 0.72 | 0.86 |
| H98 | TH151071 | L25 x T4 | 4.29 | 5.04 | . | 7.22 | 6.74 | 6.53 | 6.40 | 77 | 79 | 232 | 127 | | 1.19 | | 2.88 | 89 | | 0.83 | 0.94 |
| H99 | TH15962 | L26 x T1 | 7.46 | 6.76 | 5.92 | 7.30 | 7.27 | 4.40 | 6.52 | 76 | 77 | 258 | 137 | | 1.11 | | 2.75 | 94 | | 0.70 | 0.75 |
| H100 | VH06738 | L26 x T2 | 6.36 | 5.52 | 5.21 | 5.87 | 6.95 | 4.79 | 5.78 | 77 | 79 | 246 | 123 | | 1.05 | | 2.13 | 102 | | 0.85 | 0.83 |
| H101 | TH15965 | L26 xT3 | 6.33 | 5.33 | 4.25 | 5.87 | 4.02 | 3.52 | 4.89 | 74 | 75 | 255 | 135 | | 1.00 | | 1.63 | 96 | | 0.82 | 0.86 |
| H102 | TH15966 | L26 x T4 | 6.20 | 5.32 | 7.25 | 6.04 | 7.41 | 6.94 | 6.53 | 74 | 76 | 242 | 129 | | 1.00 | | 3.50 | 89 | | 0.77 | 0.86 |
| H103 | TH151151 | L27 x T1 | 8.02 | 8.58 | 7.09 | 6.09 | 7.31 | 5.61 | 7.12 | 75 | 76 | 261 | 148 | | 1.25 | | 2.13 | 101 | | 0.82 | 0.81 |
| H104 | VH06760 | L27 x T2 | 9.39 | 8.60 | 7.14 | 7.63 | 6.79 | 5.00 | 7.42 | 78 | 79 | 246 | 132 | | 1.35 | | 1.63 | 106 | | 0.82 | 0.77 |
| H105 | TH151153 | L27 x T3 | 6.95 | 5.94 | 5.58 | 5.96 | 5.72 | 6.28 | 6.07 | 74 | 75 | 249 | 137 | | 1.13 | | 2.00 | 99 | | 0.76 | 0.77 |
| H106 | TH151154 | L27 x T4 | 6.18 | 6.54 | 6.86 | 8.76 | 7.59 | 6.03 | 6.99 | 76 | 78 | 238 | 135 | | 1.19 | | 2.38 | 92 | | 0.85 | 0.93 |
| H107 | SC627 (non-QPM) | | 7.45 | 7.05 | 5.14 | 6.84 | 7.19 | 6.68 | 6.71 | 74 | 74 | 255 | 142 | | 0.93 | | 1.00 | 112 | | 0.57 | 0.48 |
| H108 | ZS261 (QPM) | | 6.46 | 5.58 | 5.94 | 7.14 | 5.28 | 7.03 | 6.03 | 73 | 73 | 239 | 120 | | 1.01 | | 2.00 | 105 | | 0.77 | 0.72 |
| Mean |  |  | 6.85 | 6.19 | 6.53 | 5.88 | 6.46 | 5.25 | - | 75 | 77 | 243 | 135 | | 1.13 | | 2.31 | 99 | | 0.77 | 0.78 |
| Minimum |  |  | 3.23 | 2.57 | 1.92 | 1.95 | 2.22 | 1.60 | - | 67 | 68 | 211 | 96 | | 0.92 | | 1.25 | 84 | | 0.45 | 0.44 |
| Maximum |  |  | 10.18 | 9.23 | 8.97 | 10.48 | 8.28 | 7.48 | - | 82 | 83 | 271 | 161 | | 1.60 | | 3.75 | 118 | | 0.99 | 1.01 |
| LSD |  |  | 1.80 | 1.70 | 1.94 | 3.54 | 1.72 | 1.93 | - | 2 | 2 | 10 | 8 | | 0.15 | | 0.86 | 11 | | 0.10 | 0.09 |
| NLOC |  |  | 1 | 1 | 1 | 1 | 1 | 1 | 6 | 6 | 6 | 5 | 5 | | 5 | | 2 | 2 | | 2 | 2 |
| **Local checks** | |  |  |  |  |  |  |  |  |  |  |  |  | |  | |  |  | |  |  |
|  | AMH760Q |  | - | - | - | - | - | - | - | 92 | 96 | 278 | 179 | | 0.91 | | 2.50 | - | | - | - |
|  | AMH851 |  | - | - | - | - | - | - | - | 87 | 90 | 248 | 129 | | 0.85 | | 1.00 | - | | - | - |
|  | BH546 |  | 8.82 | - | - | - | - | - | - | 75 | 78 | 287 | 142 | | 1.11 | | - | - | | - | - |
|  | BHQPY545 |  | 9.53 | - | - | - | - | - | - | 77 | 78 | 286 | 152 | | 2.20 | | - | - | | - | - |
|  | SC403 |  | - | - | 5.35 | 3.09 | 7.33 | 6.12 | - | 67 | 69 | 238 | 133 | | 0.90 | | 1.00 | 104 | | 0.53 | 0.48 |
|  | SC513 |  | - | - | 7.68 | 9.31 | 8.34 | 4.59 | - | 68 | 71 | 241 | 127 | | 0.85 | | 1.00 | 109 | | 0.60 | 0.53 |

*H107* and *H108* are non-QPM and QPM commercial checks, respectively.

*BK* Bako, *AM* Ambo, *GW* Gwebi, *GL* Glendale, *MP* Mpongwe, *CH* Chisumbanje, *AC_LOC* across all locations*, AD* days to anthesis, *DS* days to silking, *PH* plant height, *EH* ear height, *EPP* ears per plant, *MOD* kernel endosperm modification, *PRT* protein concentration, *TRP* tryptophan concentration, *QI* quality index, *NLOC* number of locations.
