# Supplementary material for: Testcross performance and combining ability of early-medium maturing quality protein maize inbred lines in Eastern and Southern Africa
Source: Sci Rep. 2024 Apr 21;14:9151. doi: 10.1038/s41598-024-58816-y (PMC11033265; doi:10.1038/s41598-024-58816-y)
Supplement: Supplementary file 2 — Supplementary Table 2. [file 41598_2024_58816_MOESM2_ESM.docx]

**Supplementary Table S2** Specific combining ability effects of 27 QPM inbred lines crossed with four testers evaluated for grain yield and other traits agronomic traits across six locations in Eastern and Southern Africa during the 2015 and 2016 cropping seasons

| Hybrid | Cross | GY | DA | DS | PH | EH | EPP | Protein |
| --- | --- | --- | --- | --- | --- | --- | --- | --- |
|  |  | t ha^-1^ | ---days-- | | ----cm--- | | --#- | ---g kg^-1^--- |
| H1 | L1 x T1 | 0.44 | -0.23 | 0.00 | 0.37 | 2.27 | -0.09 | 7.66 |
| H2 | L1 x T2 | 0.00 | 0.22 | 0.45 | 0.61 | -0.28 | 0.03 | 3.36 |
| H3 | L1 x T3 | -0.56 | 0.32 | 0.13 | -1.77 | -0.31 | -0.04 | -4.24 |
| H4 | L1 x T4 | 0.12 | -0.37 | -0.63 | 0.76 | -1.76 | 0.09 | -4.72 |
| H5 | L2 x T1 | 0.59 | 0.19 | 0.70 | 4.15 | 3.32 | -0.03 | 14.69** |
| H6 | L2 x T2 | 0.05 | -0.12 | -0.78 | -0.38 | -4.77 | 0.01 | 2.93 |
| H7 | L2 x T3 | -0.60* | 0.33 | 0.55 | -8.00** | -4.88 | 0.04 | -2.76 |
| H8 | L2 x T4 | -0.04 | -0.45 | -0.53 | 4.21 | 6.25* | -0.02 | -8.96 |
| H9 | L3 x T1 | 0.29 | 0.57 | 0.77 | -6.03 | -3.56 | 0.01 | -6.14 |
| H10 | L3 x T2 | -0.58 | -2.00** | -2.12** | 7.18* | 7.51* | -0.11 | 6.82 |
| H11 | L3 x T3 | 0.74* | 0.89 | 1.19 | -1.51 | -3.05 | 0.02 | -0.44 |
| H12 | L3 x T4 | -0.45 | 0.48 | 0.11 | 0.34 | -0.99 | 0.07 | -0.70 |
| H13 | L4 x T1 | -2.05** | 1.63** | 1.97** | -19.26** | -14.37** | -0.16** | -1.13 |
| H14 | L4 x T2 | 0.03 | -1.16* | -1.03 | -1.91 | 1.31 | 0.02 | 6.13 |
| H15 | L4 x T3 | 0.76* | -0.38 | -0.83 | 7.80* | 7.81** | 0.04 | -3.21 |
| H16 | L4 x T4 | 1.27** | -0.14 | -0.16 | 13.35** | 5.17 | 0.10 | -0.99 |
| H17 | L5 x T1 | -2.23** | 1.60** | 0.92 | -15.62** | -13.56** | 0.07 | 0.44 |
| H18 | L5 x T2 | 0.89** | 0.10 | 0.92 | 0.30 | 6.43* | 0.01 | 2.41 |
| H19 | L5 x T3 | 0.55 | -1.08** | -1.32* | 9.41** | 4.09 | -0.05 | -2.19 |
| H20 | L5 x T4 | 0.79** | -0.68 | -0.58 | 5.89 | 2.96 | -0.04 | 1.40 |
| H21 | L6 x T1 | -0.77* | 0.54 | 0.29 | -9.14** | -9.03** | -0.04 | 1.93 |
| H22 | L6 x T2 | 0.17 | 0.01 | -0.50 | 6.18 | 5.22 | -0.02 | -0.16 |
| H23 | L6 x T3 | 0.57 | -0.53 | 0.25 | 1.54 | 0.03 | 0.04 | 3.44 |
| H24 | L6 x T4 | 0.04 | -0.08 | -0.10 | 1.40 | 3.69 | 0.02 | -3.15 |
| H25 | L7 x T1 | 0.40 | -0.33 | 0.05 | 2.02 | 1.31 | -0.04 | 4.87 |
| H26 | L7 x T2 | -1.27** | 0.83 | 0.87 | -7.44* | -3.86 | 0.04 | -4.30 |
| H27 | L7 x T3 | 0.09 | 0.01 | -0.14 | 9.23** | 5.11 | 0.04 | 1.87 |
| H28 | L7 x T4 | 0.79** | -0.57 | -0.83 | -3.83 | -2.64 | -0.05 | -0.15 |
| H29 | L8 x T1 | 0.50 | -0.90 | 0.16 | -2.00 | 4.23 | 0.03 | 0.45 |
| H30 | L8 x T2 | -0.18 | 2.40** | 0.53 | -2.25 | -2.27 | 0.01 | -2.78 |
| H31 | L8 x T3 | -0.81** | -0.67 | -0.42 | 4.77 | 0.75 | -0.05 | 3.29 |
| H32 | L8 x T4 | 0.49 | -0.32 | 0.01 | -0.54 | -2.80 | 0.01 | 1.11 |
| H33 | L9 x T1 | -0.09 | -0.26 | -0.72 | 1.62 | 0.78 | -0.18** | 2.52 |
| H34 | L9 x T2 | 0.45 | 1.19* | 1.51* | 1.90 | 2.86 | 0.10 | -5.37 |
| H35 | L9 x T3 | 0.08 | -0.47 | -0.80 | 1.78 | 2.58 | 0.02 | -0.58 |
| H36 | L9 x T4 | -0.44 | -0.52 | -0.05 | -5.32 | -6.30* | 0.06 | 5.28 |
| H37 | L10 x T1 | 0.23 | 0.25 | -0.13 | 4.25 | 0.46 | -0.06 | -1.39 |
| H38 | L10 x T2 | 0.19 | -0.07 | 0.17 | 5.19 | 3.28 | 0.04 | -1.74 |
| H39 | L10 x T3 | -0.05 | -0.48 | -0.77 | -3.61 | -1.72 | 0.06 | 3.21 |
| H40 | L10 x T4 | -0.37 | 0.25 | 0.68 | -5.85 | -2.10 | -0.04 | 2.71 |
| H41 | L11 x T1 | 0.41 | -0.20 | -0.78 | 2.25 | 5.73 | 0.08 | -0.83 |
| H42 | L11 x T2 | 0.20 | -0.59 | -0.39 | 1.90 | -1.16 | -0.15** | 4.46 |
| H43 | L11 x T3 | -0.12 | 1.19* | 1.32* | 0.66 | 1.08 | 0.10 | -3.79 |
| H44 | L11 x T4 | -0.49 | -0.46 | -0.22 | -4.84 | -5.72 | -0.03 | 2.22 |
| H45 | L12 x T1 | 0.12 | -0.34 | -1.37* | 1.27 | -4.12 | -0.04 | 5.73 |
| H46 | L12 x T2 | 0.08 | -0.28 | -0.35 | -6.28 | -4.39 | -0.02 | 0.08 |
| H47 | L12 x T3 | -0.92** | 1.41** | 1.51* | 3.66 | 7.06* | 0.00 | -0.93 |
| H48 | L12 x T4 | 0.72* | -0.85 | 0.16 | 1.33 | 1.37 | 0.06 | -2.68 |
| H49 | L13 x T1 | 0.46 | -1.69** | -2.01** | 4.62 | -0.78 | 0.03 | -8.00 |
| H50 | L13 x T2 | -0.13 | -0.35 | 0.07 | -0.96 | 1.37 | -0.03 | -3.26 |
| H51 | L13 x T3 | 0.11 | 1.62** | 1.72** | 4.70 | 9.27** | 0.00 | 6.77 |
| H52 | L13 x T4 | -0.44 | 0.36 | 0.16 | -8.37* | -9.94** | 0.00 | 6.55 |
| H53 | L14 x T1 | 0.58 | -0.93 | -1.33* | -7.13* | 1.65 | 0.04 | -3.24 |
| H54 | L14 x T2 | -0.42 | 0.58 | 0.71 | 1.51 | -3.53 | -0.08 | -0.46 |
| H55 | L14 x T3 | -0.06 | -0.16 | -0.15 | -0.04 | -2.45 | 0.02 | 1.58 |
| H56 | L14 x T4 | -0.09 | 0.46 | 0.72 | 5.64 | 4.25 | 0.02 | 5.93 |
| H57 | L15 x T1 | 0.35 | -0.34 | 0.09 | -2.30 | 3.52 | 0.09 | -0.88 |
| H58 | L15 x T2 | -0.50 | -0.15 | -0.48 | 2.67 | -4.15 | -0.05 | -0.91 |
| H59 | L15 x T3 | -0.29 | 0.07 | -0.36 | -4.43 | 2.04 | -0.08 | 4.59 |
| H60 | L15 x T4 | 0.45 | 0.36 | 0.69 | 4.04 | -1.49 | 0.04 | -0.74 |

**Supplementary Table S2** Continued

| Hybrid | Cross | GY | DA | DS | PH | EH | EPP | Protein |
| --- | --- | --- | --- | --- | --- | --- | --- | --- |
|  |  | t ha^-1^ | ---days-- | | ----cm--- | | --#- | ---g kg^-1^--- |
| H61 | L16 x T1 | 0.41 | -0.31 | -0.17 | 8.18* | 4.83 | 0.02 | -3.22 |
| H62 | L16 x T2 | -0.09 | 0.25 | -0.17 | 1.79 | -1.39 | 0.18** | -2.44 |
| H63 | L16 x T3 | 0.23 | -0.35 | 0.03 | -4.77 | -1.39 | -0.11* | 2.54 |
| H64 | L16 x T4 | -0.54 | 0.36 | 0.26 | -5.21 | -2.13 | -0.10 | 5.18 |
| H65 | L17 x T1 | 0.91** | -0.18 | 0.06 | 7.39* | 10.27** | 0.09 | -0.84 |
| H66 | L17 x T2 | -0.32 | -1.22* | -1.10 | -5.90 | -10.14** | -0.07 | -6.04 |
| H67 | L17 x T3 | 0.09 | 0.10 | -0.02 | 1.59 | 0.01 | -0.02 | 5.28 |
| H68 | L17 x T4 | -0.68* | 1.26* | 1.00 | -3.11 | -0.23 | 0.01 | 4.91 |
| H69 | L18 x T3 | 0.76* | 0.58 | 0.70 | 8.14* | 4.88 | 0.04 | 1.61 |
| H70 | L18 x T4 | -0.87** | 0.58 | 0.54 | -7.64 | -2.77 | 0.01 | 0.95 |
| H71 | L19 x T1 | -0.41 | -0.54 | -0.18 | 6.27 | 4.39 | 0.13* | 5.06 |
| H72 | L19 x T2 | 0.50 | -0.19 | 0.57 | 0.82 | 0.60 | -0.05 | -5.12 |
| H73 | L19 x T3 | 0.50 | 0.03 | -0.26 | -3.55 | -10.75** | 0.05 | -5.59 |
| H74 | L19 x T4 | -0.58 | 0.65 | -0.20 | -3.56 | 5.68 | -0.13* | 8.26 |
| H75 | L20 x T1 | 0.17 | 0.91 | 0.53 | 10.42** | 7.09* | 0.02 | -2.03 |
| H76 | L20 x T2 | -0.17 | 0.61 | 1.20 | 2.30 | 9.94 | -0.03 | 0.39 |
| H77 | L20 x T3 | -0.23 | -2.41** | -2.33** | -12.48** | -18.64** | -0.04 | 2.91 |
| H78 | L20 x T4 | 0.24 | 0.84 | 0.54 | -0.26 | 1.53 | 0.05 | 2.11 |
| H79 | L21 x T1 | 0.28 | -0.09 | -0.26 | 10.93** | 8.02** | 0.06 | 1.13 |
| H80 | L21 x T2 | 0.59* | 0.95 | 0.87 | 0.29 | -0.39 | 0.06 | 2.26 |
| H81 | L21 x T3 | -0.01 | -0.79 | -0.34 | -8.67* | -5.58 | -0.05 | 0.28 |
| H82 | L21 x T4 | -0.86** | -0.12 | -0.33 | -2.57 | -2.14 | -0.07 | -1.61 |
| H83 | L22 x T1 | 0.10 | -1.08 | -0.70 | 6.50 | 0.73 | -0.08 | 2.36 |
| H84 | L22 x T2 | -0.72* | 1.49** | 1.46* | -10.09** | -3.39 | -0.07 | 1.08 |
| H85 | L22 x T3 | 1.09** | 0.33 | 0.34 | 2.15 | 0.49 | 0.12* | -0.72 |
| H86 | L22 x T4 | -0.47 | -0.38 | -0.68 | 1.42 | 2.09 | 0.03 | 0.84 |
| H87 | L23 x T1 | -0.60 | 2.87** | 3.15** | -9.58** | -5.62 | -0.03 | -2.30 |
| H88 | L23 x T2 | 0.07 | -1.04 | -1.34* | 2.73 | 3.44 | -0.02 | 6.26 |
| H89 | L23 x T3 | -0.04 | 0.11 | 0.17 | 2.35 | 1.57 | 0.01 | -0.43 |
| H90 | L23 x T4 | 0.57 | -1.48** | -1.53* | 4.48 | 0.53 | 0.04 | -1.46 |
| H91 | L24 x T1 | -1.03** | 0.56 | 0.51 | -4.37 | -3.62 | -0.11* | -5.09 |
| H92 | L24 x T2 | 0.65* | -0.05 | 0.39 | 5.16 | 0.56 | 0.12* | -0.89 |
| H93 | L24 x T3 | 0.14 | -1.11* | 1.40* | -2.64 | 0.13 | 0.02 | 9.17 |
| H94 | L24 x T4 | 0.24 | 0.54 | 0.44 | 1.84 | 2.85 | -0.03 | -3.39 |
| H95 | L25 x T1 | -0.86** | 0.38 | 0.11 | -13.34** | -7.05* | 0.03 | 0.22 |
| H96 | L25 x T2 | 0.11 | -0.80 | -0.70 | 2.57 | 6.44* | -0.04 | 4.98 |
| H97 | L25 x T3 | -0.14 | 1.72** | 1.35* | 2.20 | 1.91 | -0.02 | -5.87 |
| H98 | L25 x T4 | 0.89** | -1.19* | -0.81 | 8.55* | -1.38 | 0.02 | 1.19 |
| H99 | L26 x T1 | 0.89** | 0.40 | 0.53 | 7.34* | 0.41 | 0.07 | 2.80 |
| H100 | L26 x T2 | -0.33 | 0.62 | 1.06 | -4.02 | -4.42 | -0.04 | 1.94 |
| H101 | L26 x T3 | -0.85** | -0.05 | -0.49 | -2.83 | 1.56 | -0.01 | 0.96 |
| H102 | L26 x T4 | 0.30 | -1.03 | -1.15 | -0.51 | 2.37 | -0.02 | -3.64 |
| H103 | L27 x T1 | 0.51 | -0.94 | -0.42 | 12.25** | 4.49 | 0.01 | 5.69 |
| H104 | L27 x T2 | 0.34 | 0.97 | 0.18 | -2.84 | -3.05 | 0.07 | 1.67 |
| H105 | L27 x T3 | -0.64* | -1.00 | -0.61 | -6.74 | -3.39 | -0.07 | -0.19 |
| H106 | L27 x T4 | -0.21 | 0.92 | 0.79 | -2.68 | 1.87 | -0.02 | -5.23 |
| SE (S_ij_) |  | 0.3 | 0.54 | 0.61 | 3.46 | 2.96 | 0.05 | 5.32 |

*GY* grain yield, *AD* days to anthesis, *DS* days to silking, *PH* plant height, *EH* ear height, *EPP* ears per plant.
