# Supplementary material for: Testcross performance and combining ability of early-medium maturing quality protein maize inbred lines in Eastern and Southern Africa
Source: Sci Rep. 2024 Apr 21;14:9151. doi: 10.1038/s41598-024-58816-y (PMC11033265; doi:10.1038/s41598-024-58816-y)
Supplement: Supplementary file 3 — Supplementary Legends. [file 41598_2024_58816_MOESM3_ESM.docx]

**Supplementary Table S1** Mean performances of 106 testcross hybrids, commercial and local checks for grain yield, agronomic and protein quality traits evaluated across six locations in Southern and Eastern Africa during the 2015 and 2016 cropping seasons.

**Supplementary Table S2** Specific combining ability effects of 27 QPM inbred lines crossed with four testers evaluated for grain yield and other traits agronomic traits across six locations in Eastern and Southern Africa during the 2015 and 2016 cropping seasons.
